# Multi-omics profiling reveals molecular features of visceral adiposity in autonomous cortisol secretion

**DOI:** 10.1016/j.isci.2026.117776

**Published:** 2026-10-08

**Authors:** Hiroshi Nakao, Maki Yokomoto-Umakoshi, Masamichi Fujita, Kohta Nakatani, Yuya Kitamura, Ryo Ogasawara, Hiroki Kaneko, Norifusa Iwahashi, Yui Nakano, Tazuru Fukumoto, Tatsuki Ogasawara, Hiroki Takayanagi, Takashi Matsumoto, Yoshihiro Izumi, Masatoshi Eto, Takeshi Bamba, Hironobu Umakoshi, Yoshihiro Ogawa

**Affiliations:** 1 Department of Medicine and Bioregulatory Science, Graduate School of Medical Sciences, Kyushu University, Fukuoka, Japan; 2 Division of Metabolomics, Medical Research Center for High Depth Omics, Medical Institute of Bioregulation, Kyushu University, Fukuoka, Japan; 3 Center for Research Promotion, School of Medicine, Niigata University, Niigata, Japan; 4 Department of Urology, Graduate School of Medical Sciences, Kyushu University, Fukuoka, Japan; 5 Mass Spectrometry Center, Graduate School of Science, The University of Osaka, Toyonaka, Japan; 6 Department of Systems Metabolomics, Graduate School of Medicine, The University of Osaka, Suita, Japan

**Keywords:** visceral adiposity, ceramide, steroid metabolites, autonomous cortisol secretion, multi-omics

## Abstract

Autonomous cortisol secretion (ACS) from adrenal adenomas increases cardiometabolic risk. Visceral adiposity is a hallmark of ACS. We characterized ACS-associated visceral adiposity using multi-omics profiling of periadrenal adipose tissue and plasma. ACS exhibited higher visceral-to-subcutaneous fat ratio (VSR), adipocyte hypertrophy, and inflammation-related pathway enrichment than non-functioning adrenal tumors. RNA-seq deconvolution and immunohistochemistry indicated increased macrophage infiltration. Plasma steroid metabolomics identified 11-deoxycorticosterone (11-DOC), a mineralocorticoid metabolite, as positively associated with VSR in premenopausal ACS women independent of cortisol. The 11-DOC-associated gene module correlated with inflammation-related pathways, notably macrophage activation. Lipidomics identified ceramides as ACS discriminators, with Cer(d18:1/16:0) most strongly associated with inflammation- and lipid metabolism-related pathways. Mendelian randomization showed genetically predicted plasma Cer(d18:1/16:0) was positively associated with trunk fat mass. These findings identify circulating 11-DOC and adipose ceramide accumulation as molecular features associated with adipose inflammation and visceral adiposity in ACS, providing hypothesis-generating insights that require functional validation.

## Highlights

• Adipocyte hypertrophy and macrophage infiltration in autonomous cortisol secretion
• Plasma 11-deoxycorticosterone is associated with adipose macrophage infiltration
• Adipose ceramide (d18:1/16:0) is associated with inflammatory tissue remodeling

## Graphical abstract

**Figure:**
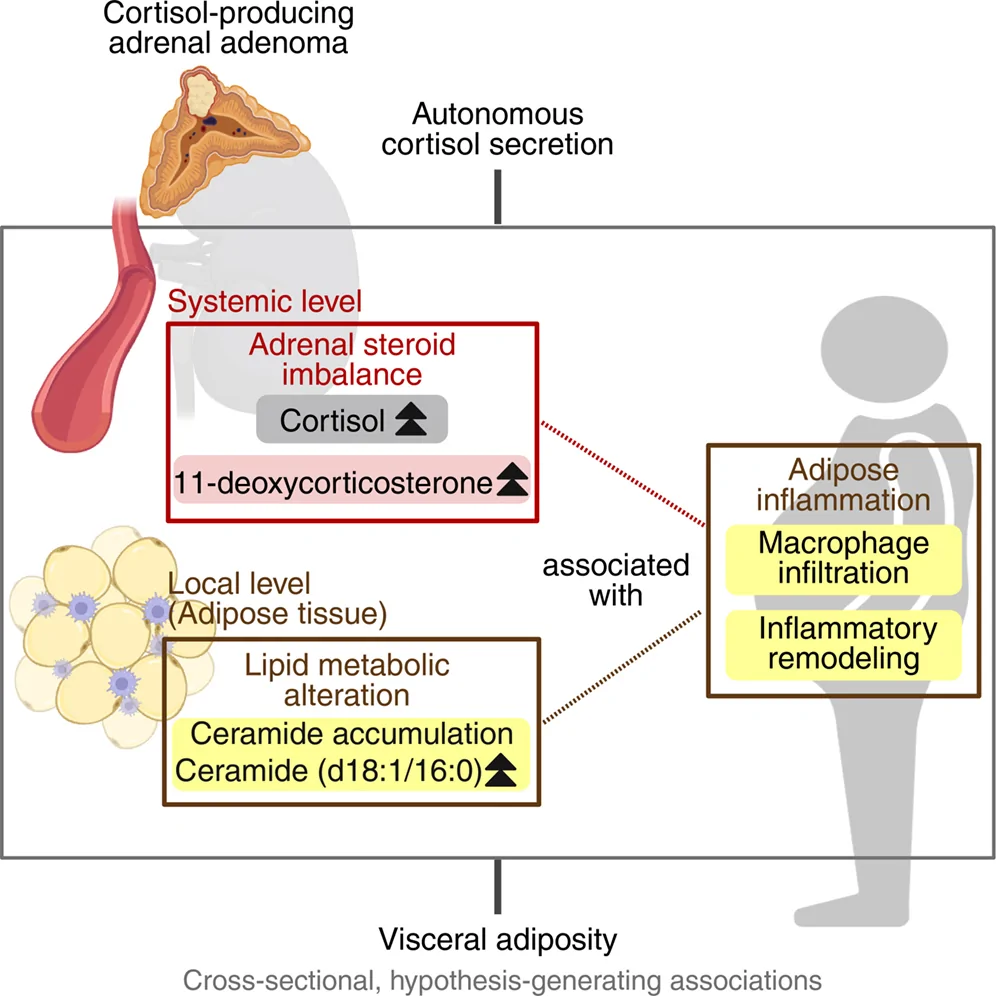

## Teaser

Health sciences

## Introduction

Cortisol-producing adrenal adenomas (CPA) give rise to autonomous cortisol secretion (ACS), which encompasses overt Cushing’s syndrome (CS) and mild autonomous cortisol secretion (MACS).^1^ While CS is relatively rare, MACS is frequently detected in adrenal incidentalomas, affecting several percent of older individuals, among which 30%–50% exhibit MACS.^2^^,^^3^ Patients with ACS frequently have metabolic diseases such as diabetes mellitus, hypertension, and dyslipidemia, which collectively contribute to increased cardiovascular morbidity and mortality.^4^^,^^5^^,^^6^ Among these complications, visceral adiposity is of particular relevance,^7^^,^^8^^,^^9^ functioning both as a central hub of metabolic dysregulation and as a key determinant of cardiovascular risk.^10^^,^^11^^,^^12^

Histological, transcriptomic, and immunohistochemical studies have reported adipose tissue remodeling in endogenous hypercortisolism, including ACS and Cushing’s disease. This remodeling, driven largely by inflammation, is characterized by adipocyte hypertrophy, macrophage infiltration, and activation of inflammatory pathways.^13^^,^^14^^,^^15^^,^^16^^,^^17^^,^^18^ Glucocorticoid receptor (GR) signaling within adipocytes has also been implicated in restricting healthy adipose tissue expansion, thereby predisposing to maladaptive adipocyte hypertrophy and inflammatory remodeling.^19^ Nevertheless, the detailed molecular mechanisms and regulatory factors underlying inflammatory remodeling in ACS remain incompletely understood.

Systemic hormonal dysregulation plays a central role in ACS, with cortisol excess influencing adipocyte differentiation, lipid metabolism, and inflammatory signaling.^20^^,^^21^ However, visceral adiposity in ACS occurs even in mild cases at levels comparable to CS,^22^ suggesting that cortisol excess alone does not fully account for this phenotype. Recent advances in mass spectrometry-based steroid profiling have revealed pathophysiological roles of circulating steroid metabolites, including their associations with cardiovascular risk in ACS.^23^^,^^24^^,^^25^ Building on these findings, we recently demonstrated, using integrated analyses of plasma and tumor tissue samples, that excess tumor-derived 11-deoxycorticosterone (11-DOC) and reduced adrenal androgens are differentially associated with adverse effects on bone metabolism independent of cortisol in ACS.^26^ These observations suggest that steroid metabolites act as complementary systemic modulators of visceral adiposity in ACS.

Beyond systemic hormonal influences, local tissue-level mechanisms may also contribute to adipose tissue dysfunction in ACS. Lipid metabolic alterations, in particular, represent an underexplored yet crucial factor: lipid species not only serve as energy substrates but also function as bioactive signaling molecules regulating adipocyte activity and inflammatory responses.^27^ However, integrated molecular profiling of human visceral adipose tissue under the endocrine milieu of ACS remains limited. In patients undergoing adrenalectomy, periadrenal adipose tissue provides an accessible visceral adipose depot for examining local tissue-level mechanisms in this context.

In this context, we hypothesized that visceral adiposity in ACS is associated with both systemic steroid hormones and local regulatory mechanisms within adipose tissue. In this study, we aimed to characterize the molecular features of visceral adiposity in human ACS through integrative profiling of clinical adiposity phenotypes, plasma steroid metabolomics, and periadrenal adipose tissue transcriptomics and lipidomics. This study was designed as an integrative, hypothesis-generating multi-omics analysis to identify systemic and tissue-level molecular features associated with visceral adiposity and adipose tissue inflammation in ACS.

## Results

### Baseline characteristics and measures of adiposity

A total of 191 patients were included in the analysis, comprising 93 patients with ACS and 98 with non-functioning adrenal tumors (NFAT) (Figure 1A; Table 1; Table S1). Patients with ACS had higher levels of serum cortisol (median interquartile range [IQR], 14.3 [10.5–18.7] μg/dL vs. 12.5 [8.7–15.8] μg/dL; *p* = 0.014), serum cortisol levels after the 1-mg dexamethasone suppression test (DST) (4.6 [2.6–12.8] μg/dL vs. 1.0 [0.7–1.3] μg/dL; *p* < 0.001), midnight cortisol, 24-h urinary free cortisol (UFC), and hemoglobin A1c than those with NFAT. Patients with ACS also had lower levels of plasma adrenocorticotropic hormone (ACTH) and dehydroepiandrosterone-sulfate (DHEA-S). There were no significant differences between the two groups in age, sex, body mass index (BMI), smoking status, alcohol intake, total cholesterol, triglyceride, or low-density lipoprotein cholesterol.

**Figure 1:**
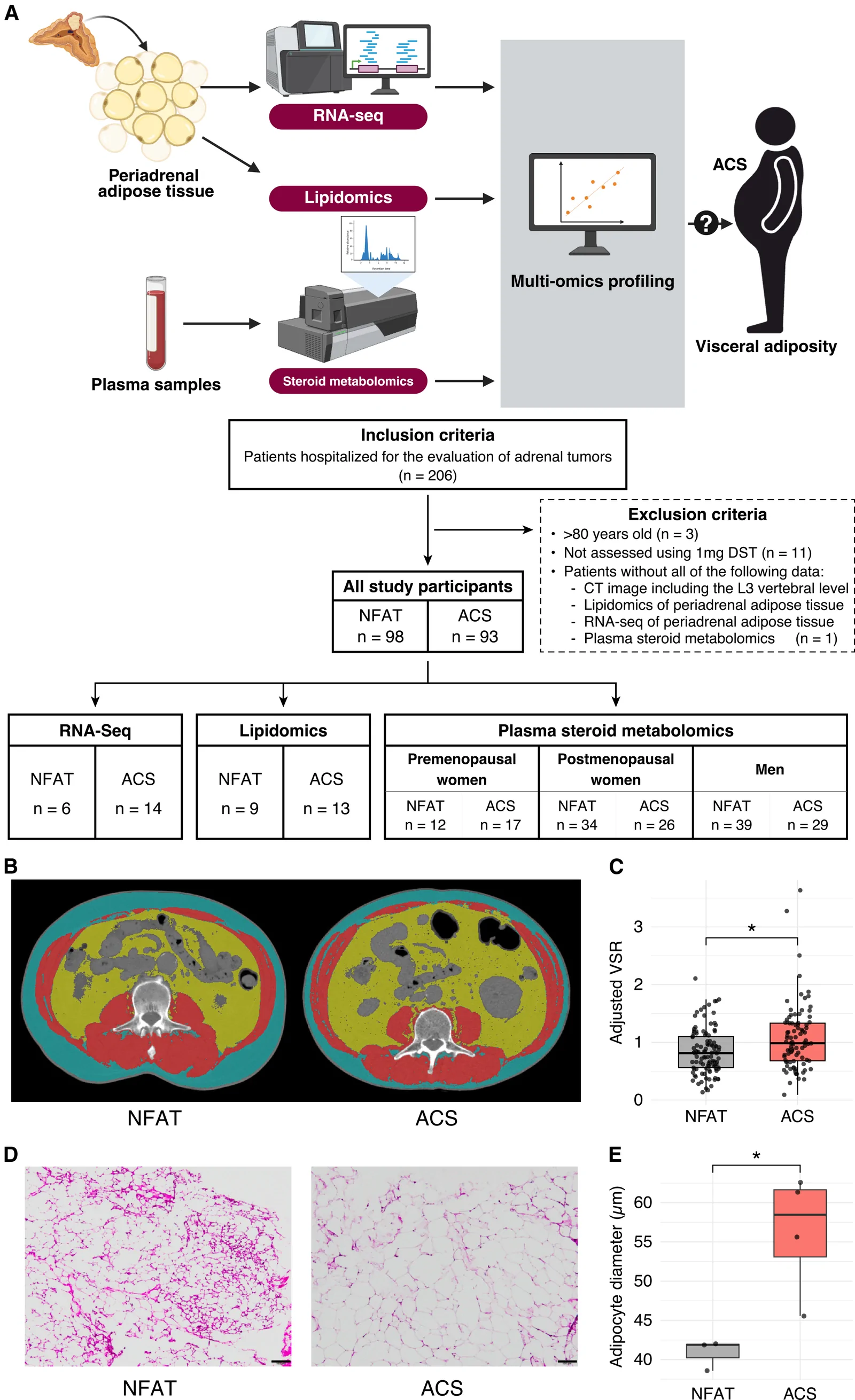
Study flow and measures of adiposity (A) Schematic of the study design and flowchart outlining inclusion/exclusion criteria and the analytic subsets. (B) Representative CT images at the L3 levels, highlighting visceral fat (yellow), subcutaneous fat (light blue), and skeletal muscle (red). (C) VSR in ACS (*n* = 92) and NFAT (*n* = 98). Boxes indicate the interquartile range (IQR; Q1–Q3), center lines indicate the median, whiskers extend to the most extreme values within 1.5×IQR, and points represent individual observations. The *p* value was calculated using a multiple linear regression model of log-transformed VSR adjusted for age, sex, BMI, smoking status, and alcohol intake (∗, *p* < 0.05). (D) Representative hematoxylin-eosin staining of periadrenal adipose tissue. Scale bars, 100 μm. (E) Adipocyte diameter in ACS (*n* = 4) and NFAT (*n* = 3). Boxes indicate the IQR, center lines indicate the median, whiskers extend to the most extreme values within 1.5×IQR, and points represent individual samples. *p* values were calculated using the Mann-Whitney U test (∗, *p* < 0.05). DST, dexamethasone suppression test; ACS, autonomous cortisol secretion; NFAT, non-functioning adrenal tumors; VSR, visceral-to-subcutaneous fat ratio; BMI, body mass index.

**Table 1:** Baseline characteristics in patients with ACS and NFAT

	NFAT		ACS		*p* value
	*n* = 98		*n* = 93 (CS = 18/MACS = 75)		
Sex = women (%)	50	(51.0)	57	(61.3)	0.199
Age (years)	59	[51, 67]	61	[46, 69]	0.769
BMI (kg/m2)	23.8	[22.1, 27.2]	24.2	[21.3, 27.2]	0.611
HbA1c (%)	5.8	[5.5, 6.2]	6.0	[5.7, 7.0]	0.001
Smoking status (%)	44	(44.9)	50	(53.8)	0.280
Alcohol intake (%)	41	(41.8)	43	(46.2)	0.641
ACTH (pg/mL)	24.7	[17.4, 43.3]	6.2	[1.5, 15.0]	<0.001
Cortisol (μg/dL)	12.5	[8.7, 15.8]	14.3	[10.5, 18.7]	0.014
DHEA-S (μg/dL)	93	[61, 150]	38	[16, 65]^#1^	<0.001
DST-cortisol (μg/dL)	1.0	[0.7, 1.3]	4.6	[2.6, 12.8]	<0.001
Midnight cortisol (μg/dL)	2.8	[2.0, 4.2]^#1^	6.6	[4.9, 12.5]^#2^	<0.001
24-h UFC (μg/day)	44.3	[31.9, 61.6]^#8^	64.9	[38.6, 105.0]^#4^	<0.001
TC (mg/dL)	194	[170, 220]^#1^	203	[177, 225]	0.140
TG (mg/dL)	130	[91, 174]	118	[86, 170]	0.457
HDL-C (mg/dL)	49	[42, 66]	53	[44, 70]^#2^	0.040
LDL-C (mg/dL)	108	[95, 126]	114	[90, 131]^#1^	0.712

Numbers of missing values are indicated as superscripts next to the interquartile range (e.g., #1).

ACS, autonomous cortisol secretion; NFAT, non-functioning adrenal tumors; CS, Cushing’s syndrome; MACS, mild autonomous cortisol secretion; BMI, body mass index; HbA1c, hemoglobin A1c; ACTH, adrenocorticotropic hormone; DHEA-S, dehydroepiandrosterone-sulfate; DST, dexamethasone suppression test; 24-h UFC, 24-h urinary free cortisol.

Body composition was evaluated using axial computed tomography (CT) images (ACS, *n* = 92; NFAT, *n* = 98), and visceral-to-subcutaneous fat ratio (VSR) was calculated (Figure 1B). Patients with ACS showed a significantly higher VSR than those with NFAT after adjustment for age, sex, BMI, smoking status, and alcohol intake (adjusted mean, 0.98 [0.68–1.33] and 0.81 [0.56–1.10]; *p* = 0.001) (Figure 1C), suggesting increased visceral adiposity. Histological analysis of periadrenal adipose tissue using hematoxylin and eosin (HE)-stained sections (ACS, *n* = 4; NFAT, *n* = 3) (Figure 1D) showed larger adipocytes in ACS than in NFAT (58.5 μm [53.1–61.6] and 41.9 μm [40.2–42.0]; *p* = 0.03) (Figure 1E), consistent with adipocyte hypertrophy in ACS.

### Transcriptomic characterization of periadrenal adipose tissue

To investigate the molecular characteristics of visceral adiposity in ACS, we performed bulk RNA-seq on periadrenal adipose tissue (ACS, *n* = 14; NFAT, *n* = 6). Uniform manifold approximation and projection (UMAP) visualization revealed distinct transcriptomic profiles between ACS and NFAT groups (Figure 2A), suggesting clear molecular differences in adipose tissue. Differential gene expression analysis identified 110 upregulated and 41 downregulated genes in ACS (Table S2). Upregulated genes included those related to inflammatory signaling (*NFKBIA*, *PTX3*, and *SERPINE1*), stress response (*GADD45G*, *MT1M*, and *MT2A*), metabolic reprogramming (*PDK4*), and immune regulation (*MERTK* and *VEGFA*) (Figure 2B). Using decoupleR (version 2.10.0),^28^ we found elevated mitogen-activated protein kinase (MAPK) and tumor necrosis factor (TNF)-α signaling in ACS (Figure 2C). Consistently, transcription factors such as AP-1 (FOS and JUN), downstream of MAPK, and nuclear factor (NF)-KB were activated (Figure 2D). Pathway enrichment analysis further showed enrichment of inflammatory pathways, including TNF-α signaling via NF-kB, regulation of the p38MAPK cascade, and regulation of phagocytosis. Notably, the ceramide signaling pathway was also enriched, suggesting involvement of lipid-mediated signaling in adipose tissue inflammation (Figure 2E).

**Figure 2:**
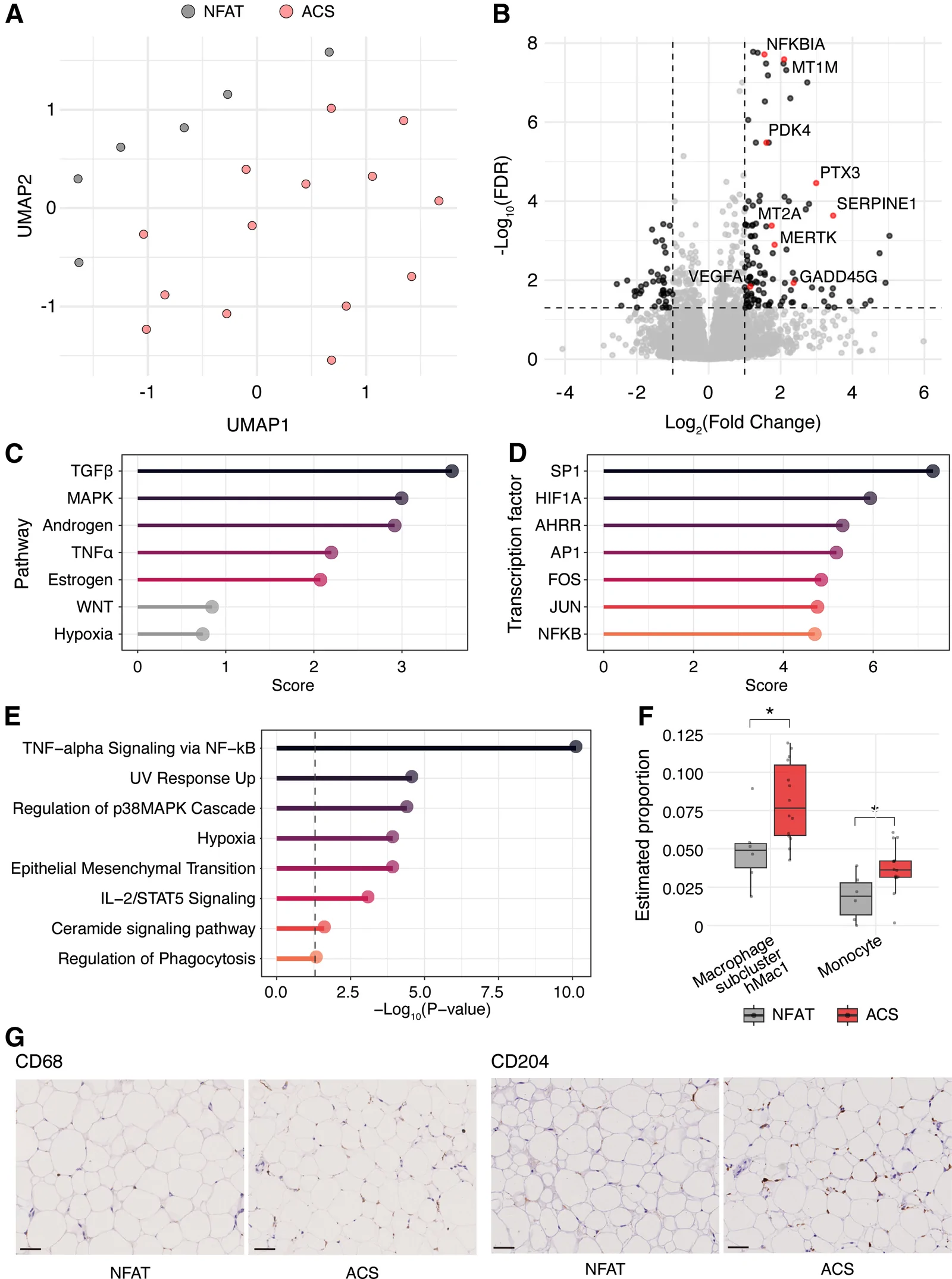
Transcriptomic characterization of periadrenal adipose tissue (A) UMAP visualization of RNA-seq data (ACS, *n* = 14; NFAT, *n* = 6). (B) Volcano plot showing log2 fold changes and statistical significance of genes differentially expressed between ACS and NFAT groups (|log2FC| > 1 and FDR < 0.05). Genes upregulated in ACS are shown on the right; downregulated genes are shown on the left. Key upregulated genes are highlighted in red. (C) Top seven signaling pathways ranked by inferred pathway activity. (D) Top seven transcription factors ranked by inferred activity from gene expression patterns. (E) Pathway enrichment analysis of upregulated DEGs. Representative enriched pathways were selected based on statistical significance and biological relevance to inflammation and adipose tissue remodeling. The dashed line indicates *p* = 0.05. (F) Estimated proportions of monocytes and macrophage subcluster hMac1 inferred by deconvolution using scRNA-seq reference data (ACS, *n* = 14; NFAT, *n* = 6). Boxes indicate interquartile ranges, horizontal lines indicate medians, and dots indicate individual samples. *p* values were calculated using the Mann-Whitney U test (∗, *p* < 0.05). (G) Representative images of CD68 and CD204 immunohistochemistry in FFPE periadrenal adipose tissue sections from ACS cases (*n* = 6) and one available NFAT case. Scale bars, 50 μm. ACS, autonomous cortisol secretion; NFAT, non-functioning adrenal tumors; UMAP, uniform manifold approximation and projection; DEG, differentially expressed genes; FDR, false discovery rate.

We next compared our adipose tissue signature with the RNA sequencing (RNA-seq) dataset from García-Eguren et al.,^15^ which includes omental visceral adipose tissue from CS (*n* = 6) and matched controls (*n* = 12). Among 50 MSigDB Hallmark gene sets, 40 showed concordant directionality between our ACS vs. NFAT signature and the CS vs. control signature, including 15 that were positively enriched in both datasets at adjusted *p* < 0.05 (Figure S1A; Table S3). Because our ACS group included both CS and MACS, we further restricted the analysis to MACS cases (*n* = 9) and found 10 gene sets positively enriched in both the MACS vs. NFAT and overt CS vs. control comparisons. These shared gene sets were mainly related to inflammatory and immune responses.

In addition, deconvolution analysis using visceral adipose tissue-derived reference cells showed higher estimated proportions of macrophage subcluster hMac1, characterized by *MRC1*, *CD163L1*, and *LYVE1* expression in the reference dataset, and monocytes in ACS than in NFAT (hMac1, 0.077 [0.059–0.105] vs. 0.049 [0.038–0.053], *p* = 0.017; monocytes, 0.036 [0.031–0.042] vs. 0.019 [0.007–0.028], *p* = 0.021) (Figure 2F; Figure S1B; Table S4). Among non-immune cell types, ACS showed a lower estimated adipocyte proportion and a higher lymphatic endothelial cell proportion than NFAT (Figure S1B; Table S4). Immunohistochemistry of formalin-fixed paraffin-embedded (FFPE) periadrenal adipose tissue from ACS (*n* = 6) showed widespread distribution of the macrophage markers CD68^−^and CD204 (encoded by *MSR1*)-positive cells (Figure 2G). In a quantitative analysis using optimal cutting temperature compound (OCT)-embedded frozen sections, CD204-positive cell density was higher in ACS than in NFAT (median [IQR], 44.8 [36.7, 53.1] vs. 29.5 [21.4, 31.0] cells/mm^2^; *p* = 0.033; Figure S1C).

### Association between plasma steroid metabolites and visceral adiposity

We next evaluated the correlation between 21 plasma steroid metabolites and VSR (Figure 3A). Patients were stratified into premenopausal women (ACS, *n* = 17; NFAT, *n* = 12), postmenopausal women (ACS, *n* = 26; NFAT, *n* = 34), and men (ACS, *n* = 29; NFAT, *n* = 39). Among all steroid metabolites, 11-DOC, a mineralocorticoid metabolite which we previously reported to be elevated in ACS,^26^ was positively correlated with VSR in premenopausal women with ACS (R = 0.63, false discovery rate [FDR] = 0.05) (Figures 3B and 3C; Table S5). This association remained significant after adjusting for serum cortisol, BMI, smoking status, and alcohol intake (estimate, 0.08, standard error [SE], 0.03, *p* = 0.04; Table S6). However, in a sensitivity analysis using the overall ACS cohort, 11-DOC was not significantly associated with VSR after adjustment for age, sex, BMI, serum cortisol, smoking status, and alcohol intake (estimate, 0.09; SE, 0.10; *p* = 0.41; Table S6). A few other associations reached FDR significance in other ACS subgroups, but none remained significant after covariate adjustment. No associations were observed in any NFAT subgroups (Table S7).

**Figure 3:**
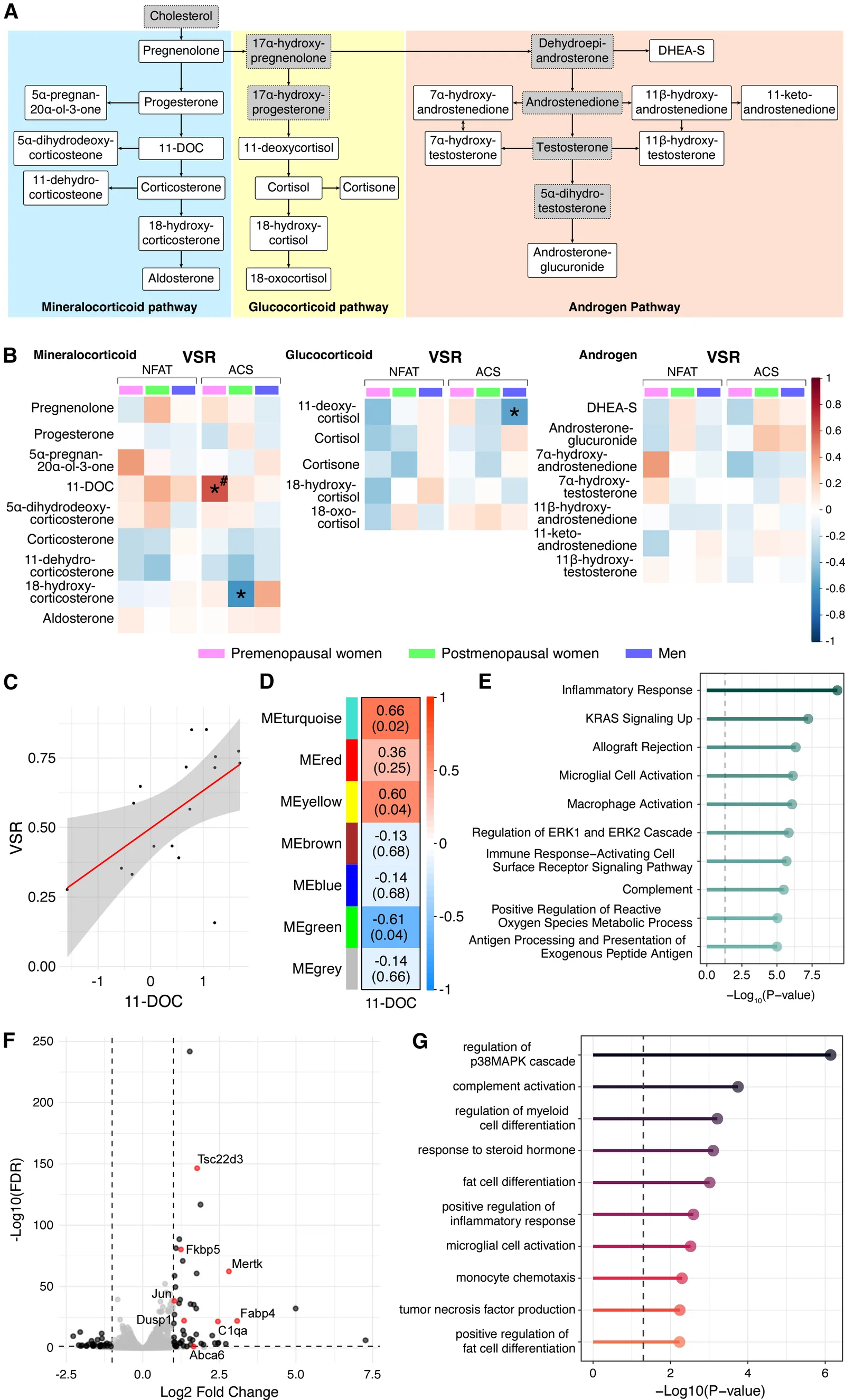
Association between plasma steroid metabolites and visceral adiposity (A) Pathway diagram of mineralocorticoids, glucocorticoids, and androgens. Steroid metabolites measured in this study are highlighted. (B) Correlation matrix showing associations between plasma steroid metabolites and VSR in ACS and NFAT. Patients were stratified into premenopausal women (ACS, *n* = 17; NFAT, *n* = 12), postmenopausal women (ACS, *n* = 26; NFAT, *n* = 34), and men (ACS, *n* = 29; NFAT, *n* = 39). Correlations were assessed using Spearman’s rank correlation (∗, Benjamini-Hochberg FDR < 0.1); #, significance in a multiple linear regression model adjusted for serum cortisol, BMI, smoking status, and alcohol intake. (C) Scatterplot of plasma 11-DOC levels and VSR in premenopausal women with ACS. The red line indicates the linear regression fit, and the shaded area indicates its 95% confidence interval. (D) Heatmap showing correlations between WGCNA modules and plasma 11-DOC levels in paired metabolomics-RNA-seq data (ACS, *n* = 10; NFAT, *n* = 2). Each cell shows the correlation coefficient and *p* value. Red and blue colors indicate positive and negative correlations, respectively, with color intensity reflecting correlation strength. (E) Pathway enrichment analysis of genes in the turquoise WGCNA module (MEturquoise), which showed the strongest positive correlation with plasma 11-DOC levels. Representative enriched pathways were selected based on statistical significance and biological relevance to inflammation and macrophage activation. The dashed line indicates *p* = 0.05. (F) Volcano plot of DEG in RAW264 macrophages treated with 11-DOC and control (|logFC| > 1 and FDR <0.05). Key upregulated genes are highlighted in red. (G) Pathway enrichment analysis of upregulated genes from *in vitro* RNA-seq. Representative enriched pathways are shown. ACS, autonomous cortisol secretion; NFAT, non-functioning adrenal tumors; 11-DOC, 11-deoxycorticosterone; DHEA-S, dehydroepiandrosterone-sulfate; VSR, visceral-to-subcutaneous fat ratio; WGCNA, weighted gene co-expression network analysis; ME, module eigengene; FDR, false discovery rate. The dashed line indicates *p* = 0.05.

To explore transcriptional correlates of plasma 11-DOC levels, we performed weighted gene co-expression network analysis (WGCNA, version 1.72.5)^29^^,^^30^ using samples with matched RNA-seq and steroid metabolomics data (ACS, *n* = 10; NFAT, *n* = 2). Among seven identified gene modules, the turquoise module (189 genes, including *CD163*, *MSR1*, *MERTK*, *CTSS*, and *FCGR2A*) showed the strongest positive correlation with plasma 11-DOC levels (R = 0.66, *p* = 0.02) (Figures 3D; Table S8). Leave-one-out sensitivity analysis showed that the correlation between MEturquoise and plasma 11-DOC remained consistently positive after exclusion of each individual sample (r = 0.57–0.78; Table S9), suggesting that this association was not driven by a single sample. This module was enriched in inflammation- and immune-related pathways, such as inflammatory response and macrophage activation (Figures 3E; Table S10). These findings indicate that plasma 11-DOC levels are associated with adipose tissue gene modules related to immune activation, inflammation, and tissue remodeling.

We performed RNA-seq analysis of RAW264 cells treated with 11-DOC (1,000 nM, 48 h) to examine macrophage transcriptional responses to pharmacological 11-DOC exposure. A total of 50 genes were upregulated and 29 were downregulated in 11-DOC-treated cells. Upregulated genes included *Fabp4*, *Mertk*, *Jun*, *C1qa*, and *Fkbp5* (Figures 3F; Table S11). A gene-level comparison with the human 11-DOC-associated turquoise module identified five overlapping genes among the 11-DOC-upregulated genes in RAW264 cells (*C1qa*, *C1qb*, *C1qc*, *Ms4a4a*, and *Stab1*; Table S11). Pathway enrichment analysis showed significant enrichment of fat cell differentiation, complement activation, regulation of p38 MAPK cascade, and response to steroid hormone (Figures 3G; Table S12). To evaluate whether the transcriptional changes were accompanied by overt cytotoxicity, we performed a Cell Counting Kit-8 (CCK-8) assay under the same exposure conditions used for RNA-seq. Treatment with 1,000 nM 11-DOC for 48 h did not significantly reduce cell viability at either cell density or any detection time tested (Figure S2A). We also performed quantitative polymerase chain reaction (qPCR) validation of representative genes upregulated in the RNA-seq analysis. The increased expression of *Mertk*, *C1qa*, and *Fkbp5* was confirmed after 1,000 nM 11-DOC treatment for 48 h (Figure S2B). In a dose-response qPCR analysis, *Mertk* expression differed significantly across 11-DOC concentrations and was increased at 1,000 nM, but not at 100 nM, compared with control (Figure S2C). Thus, pharmacological 11-DOC exposure was associated with transcriptional changes related to lipid-related metabolism, immune-related response, and steroid hormone response.

### Lipidomic characterization of periadrenal adipose tissue

To characterize lipid-metabolic alterations in ACS, we performed lipidomics on periadrenal adipose tissue (ACS, *n* = 13; NFAT, *n* = 9). Among the total lipid profile (18 subclasses across 5 lipid classes), ACS had higher total ceramide (Cer) levels, a major sphingolipid, than NFAT (log fold change [logFC] = 0.42, FDR = 0.007; Figure 4A; Table S13). Partial least squares-discriminant analysis (PLS-DA) based on total lipid profiles showed separation between ACS and NFAT groups (Figure 4B), with ceramide exhibiting the highest variable importance in projection (VIP) score (VIP = 2.22) (Table S14).

**Figure 4:**
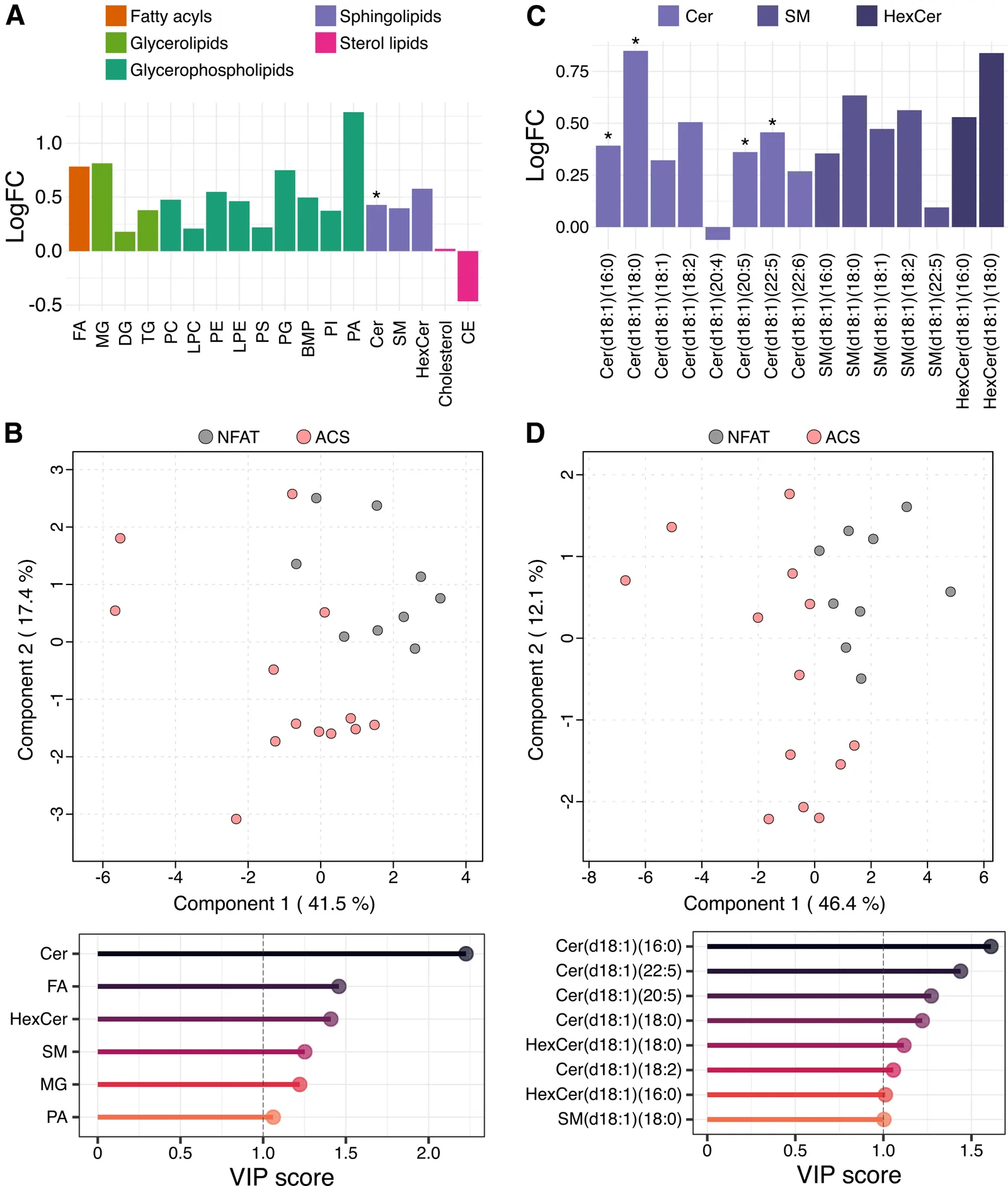
Lipidomic characterization of periadrenal adipose tissue (A) Total lipid class concentrations in ACS (*n* = 13) and NFAT (*n* = 9). Positive logFC values indicate higher levels in ACS, and negative values indicate lower levels in ACS. Group comparisons were performed using a two-tailed Student’s *t* test on the normalized data (∗, Benjamini-Hochberg FDR < 0.1). (B) PLS-DA of total lipid class concentrations. Lipid classes with VIP scores >1, indicating above-average contribution to group discrimination in the PLS-DA model, are shown below. (C) Individual sphingolipid species in ACS and NFAT. Positive logFC values indicate higher levels in ACS, and negative values indicate lower levels in ACS. Group comparisons were performed using a two-tailed Student’s *t* test on the normalized data (∗, Benjamini-Hochberg FDR < 0.1). (D) PLS-DA of individual sphingolipid species. Species with VIP scores >1, indicating above-average contribution to group discrimination in the PLS-DA model, are shown below. FA, fatty acyls; MG, monoacylglycerols; DG, diacylglycerols; TG, triacylglycerols; PC, phosphatidylcholine; LPC, lysophosphatidylcholine; PE, phosphatidylethanolamine; LPE, lysophosphatidylethanolamine; PS, phosphatidylserine; PG, phosphatidyl-glycerol; BMP, bis(monoacylglycero)phosphate; PI, phosphatidylinositol; PA, phosphatidic acid; Cer, ceramide; SM, sphingomyelin; HexCer, hexosylceramide; CE, cholesteryl esters; FC, fold change; ACS, autonomous cortisol secretion; NFAT, non-functioning adrenal tumors; VIP, variable importance in projection; FDR, false discovery rate; PLS-DA, partial least squares-discriminant analysis.

Focusing on sphingolipids, ACS had higher levels of multiple ceramide species—Cer(d18:1/16:0) (logFC = 0.39, FDR = 0.01), Cer(d18:1/18:0), Cer(d18:1/20:5), and Cer(d18:1/22:5)—than NFAT (Figures 4C; Table S15). PLS-DA based on sphingolipid profiles also distinguished ACS from NFAT, with Cer(d18:1/16:0) showing both the lowest FDR and the highest VIP score (VIP = 1.61), emerging as the most prominent candidate lipid species associated with ACS (Figures 4D; Table S16). While analyses of the other four lipid classes (fatty acyls [FA], glycerolipids, glycerophospholipids, and sterol lipids) identified several species differing between ACS and NFAT, these categories did not show the clear PLS-DA separation observed with sphingolipids (Figures S3A–S3D; Table S17). Among fatty acyl species, FA(16:0), corresponding to palmitic acid and the C16:0 acyl chain of Cer(d18:1/16:0), was increased in ACS compared with NFAT (logFC = 0.843, *p* = 0.020, FDR = 0.081; Figure S3A; Table S17).

### Association between ceramide accumulation, adipose inflammation, and adiposity

To explore transcriptional programs linked to Cer(d18:1/16:0) accumulation, we performed WGCNA (version 1.72.5)^29^^,^^30^ using periadrenal adipose tissue with matched RNA-seq and lipidomics data (ACS, *n* = 11; NFAT, *n* = 5). Among six identified gene modules, the indigo module (46 genes, including *NFKBIA*, *PTX3*, *GADD45G*, *VEGFA*, and *PDK4*) showed the strongest positive correlation with Cer(d18:1/16:0) levels (R = 0.81, *p* < 0.01) (Figure 5A; Table S18). Leave-one-out sensitivity analysis showed that the Cer(d18:1/16:0)-associated indigo module remained positively correlated in 15 of 16 iterations (r = −0.15 to 0.88) but reached statistical significance in only 9 of 16 iterations (*p* < 0.05). Unlike the 11-DOC-associated turquoise module, which remained stable across all iterations, this ceramide-associated module was therefore more sensitive to the exclusion of individual samples (Table S19). This module was enriched in pathways related to inflammation and stress, including the TNF-α signaling via NF-kB, hypoxia, vasculogenesis, and the p38 MAPK cascade, as well as pathways related to adipose tissue development and fatty acid metabolism (Figures 5B; Table S20). These findings suggest that ceramide accumulation may be related to inflammatory remodeling and metabolic reprogramming of adipose tissue.

**Figure 5:**
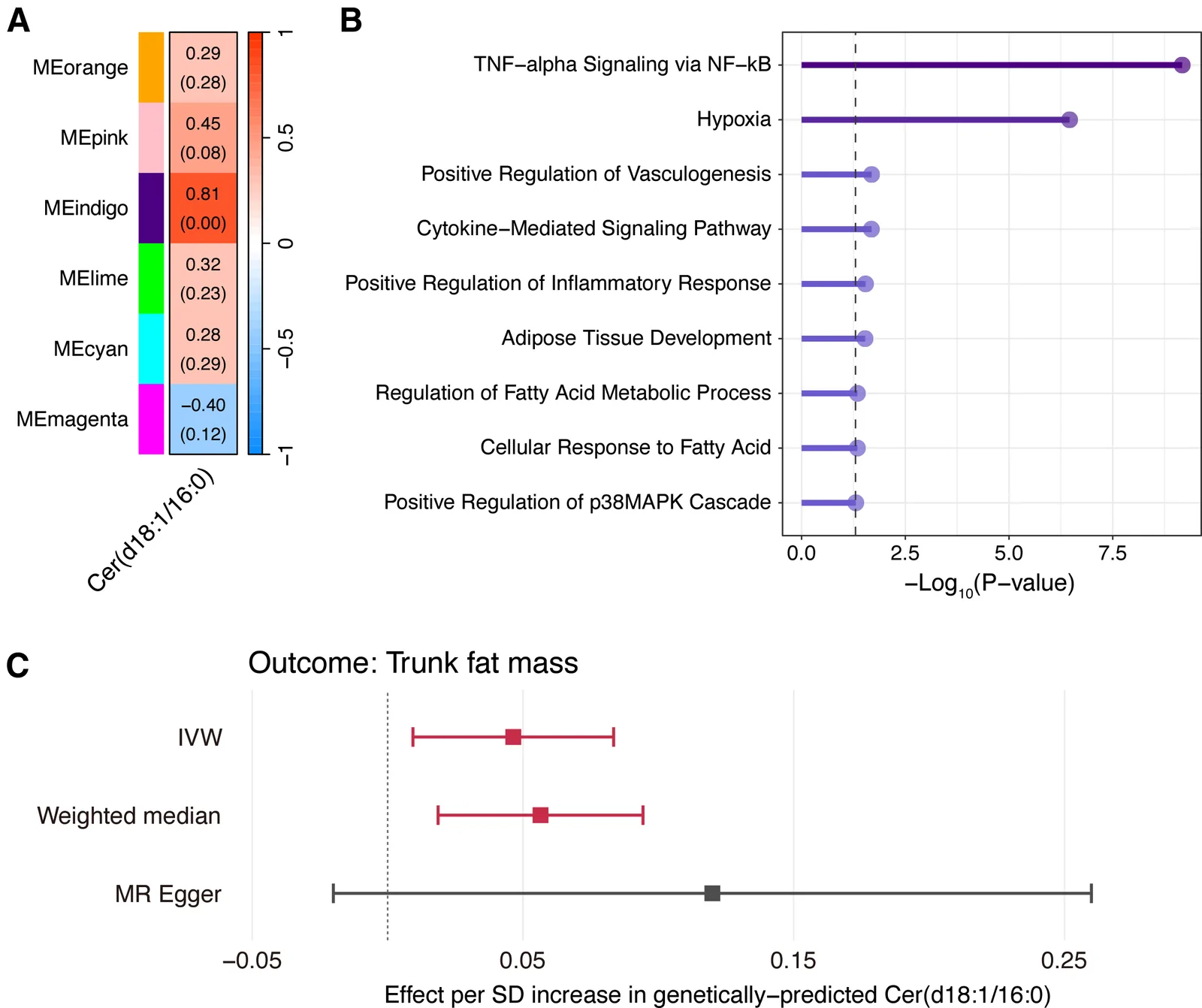
Association between ceramide accumulation, adipose inflammation, and adiposity (A) Heatmap showing correlations between WGCNA modules and Cer(d18:1/16:0) levels in paired lipidomics-RNA-seq data (ACS, *n* = 11; NFAT, *n* = 5). Each cell shows the correlation coefficient and *p* value. Red and blue colors indicate positive and negative correlations, respectively, with color intensity reflecting correlation strength. (B) Pathway enrichment analysis of genes in the indigo WGCNA module (MEindigo), which showed the strongest positive correlation with Cer(d18:1/16:0) levels. Representative enriched pathways were selected based on statistical significance and biological relevance to inflammation, stress response, and lipid metabolism. The dashed line indicates *p* = 0.05. (C) Forest plot of Mendelian randomization analysis for plasma Cer(d18:1/16:0) levels and trunk fat mass (after outlier correction using MR-PRESSO). Points indicate effect estimates and error bars represent 95% confidence intervals. ACS, autonomous cortisol secretion; NFAT, non-functioning adrenal tumors; IVW, inverse-variance-weighted; MR, Mendelian randomization; SD, standard deviation; WGCNA, weighted gene co-expression network analysis; ME, module eigengene.

To assess whether the 11-DOC-associated turquoise module (Figure 3D) and the Cer(d18:1/16:0)-associated indigo module reflected a shared transcriptional program, we compared their gene composition. Only two genes (*ARRDC2* and *PLA2G2A*) were shared between the two modules, suggesting that 11-DOC and Cer(d18:1/16:0) are associated with largely distinct transcriptional programs rather than a single shared signature.

Finally, to assess the broader biological significance of Cer(d18:1/16:0) beyond local adipose tissue effects, we performed a two-sample Mendelian randomization analysis using European-ancestry genome-wide association study (GWAS) datasets.^31^^,^^32^ Six single nucleotide polymorphisms (SNPs) associated with plasma Cer(d18:1/16:0) levels were used as instrumental variables (Table S21), with trunk fat mass as the outcome. Genetically predicted plasma Cer(d18:1/16:0) was positively associated with trunk fat mass in both the inverse-variance weighted (IVW) and weighted median analyses using all SNPs (Figure S4A; Table S21). MR-Egger analysis did not indicate potential directional pleiotropy (*p* > 0.05), whereas Cochran’s Q test suggested heterogeneity (*p* < 0.05). Leave-one-out analysis showed consistently positive effect estimates, although statistical significance was lost after removal of some individual SNPs (Figure S4B; Table S21). Given the heterogeneity detected in the all-SNP analysis, MR-PRESSO was applied and identified three outlier SNPs. Phenome-wide association study (PheWAS) analysis showed that two of these outlier SNPs had non-lipid trait associations potentially relevant to horizontal pleiotropy (Table S21). After excluding the outliers, the positive association remained significant in both IVW analysis (β = 0.046, 95% confidence interval [CI] = 0.009–0.083, *p* = 0.014) and weighted median analysis (β = 0.056, 95% CI = 0.018–0.094, *p* = 0.003), and heterogeneity was no longer detected (Figures 5C; Table S21).

## Discussion

This study used a multi-omics approach to characterize molecular features of visceral adiposity in ACS. Consistent with previous studies,^13^^,^^14^^,^^15^^,^^16^^,^^17^^,^^18^ ACS was associated with increased visceral adiposity, adipocyte hypertrophy, inflammatory pathway enrichment, and macrophage-related RNA signals in periadrenal adipose tissue. By integrating plasma steroid metabolomics and adipose tissue lipidomics with transcriptomic data, we identified two molecular features associated with adipose inflammation: circulating 11-DOC and adipose ceramide accumulation. We previously showed that CPA tissue exhibits increased 11-DOC production through elevated CYP21A2 expression and that plasma 11-DOC is associated with bone loss independent of cortisol.^26^ The present study extends this concept to adipose tissue, linking 11-DOC to visceral adiposity and macrophage-related inflammatory gene modules. Together with recent evidence that intratumoral heterogeneity in steroidogenesis influences clinical outcomes in aldosterone-producing adenomas,^33^ these findings support the broader concept that imbalanced adrenal steroid production contributes to tissue-specific complications across adrenocortical tumors.

In this study, plasma 11-DOC was associated with macrophage-related inflammatory gene modules in ACS adipose tissue, and RAW264 cell experiments were performed as an *in vitro* follow-up of this finding. 11-DOC binds to the mineralocorticoid receptor (MR) with an affinity similar to aldosterone, exhibiting approximately 70% of its activity.^34^^,^^35^ Both macrophages and adipocytes express GR and MR,^21^^,^^36^^,^^37^ and low expression of 11β-hydroxysteroid dehydrogenase type 2 may permit steroid-dependent MR signaling.^21^^,^^36^^,^^37^^,^^38^ MR signaling has been implicated in pro-inflammatory and tissue-remodeling processes, whereas GR signaling exerts more context-dependent effects.^39^ Aldosterone also activates macrophages, and macrophage MR signaling contributes to inflammation, tissue remodeling, obesity, and metabolic dysfunction.^38^^,^^40^^,^^41^^,^^42^^,^^43^ Mineralocorticoids may also cooperate with glucocorticoids to activate MR in adipocytes,^44^ raising the possibility that 11-DOC may also be related to adipocyte responses. Taken together, previous studies on mineralocorticoid signaling provide a biological context for our findings and suggest a plausible link between 11-DOC and macrophage-related inflammatory programs in adipose tissue. However, the specific receptor pathway mediating the effect of 11-DOC was not experimentally defined in this study. Thus, the RAW264 data should be viewed as a simplified, hypothesis-generating *in vitro* follow-up of the macrophage-related signatures observed in human ACS adipose tissue, rather than as direct proof of adipose tissue inflammation *in vivo*.

The subgroup-specific association between 11-DOC and VSR in premenopausal women may reflect differences in sex-hormone milieu and adipose tissue phenotype. Estrogen influences visceral fat distribution and adipose tissue remodeling, and estrogen receptor signaling can modulate MR transcriptional activity.^45^^,^^46^ Progesterone and its metabolites may also interact with the human MR in a context-dependent manner.^35^ These factors, together with menopausal and age-related metabolic changes, may have made the 11-DOC-VSR association more apparent in premenopausal women and less evident in postmenopausal women and men.

Our adipose tissue lipidomics revealed distinct lipid profiles between ACS and NFAT, with ceramides representing the primary discriminating lipid class elevated in ACS; RNA-seq also showed ceramide pathway enrichment. Ceramides accumulate under metabolic stress and are implicated in insulin resistance and inflammation.^47^ The ceramide accumulation observed in ACS aligns with experimental evidence that chronic glucocorticoid exposure promotes ceramide synthesis and metabolic abnormalities.^48^ Among ceramide species, Cer(d18:1/16:0), a C16:0 ceramide containing palmitic acid, showed the strongest association with ACS. C16:0 ceramide can be generated in adipocytes and macrophages in a CerS5/6-dependent manner.^49^^,^^50^^,^^51^ Given these prior findings and the inflammatory signatures observed in our ACS adipose tissue samples, local immune-adipocyte interactions and lipid metabolic remodeling may contribute to ceramide accumulation in ACS. Although adipose ceramide accumulation has been reported in obesity,^52^ BMI did not differ significantly between ACS and NFAT groups, suggesting ACS-associated lipid remodeling rather than simple obesity-related changes. Consistently, Cer(d18:1/16:0) was positively correlated with inflammatory and lipid-metabolic gene modules in ACS adipose tissue, in line with studies showing that adipose or macrophage-derived C16:0 ceramide promotes metabolic dysfunction and inflammation-related signaling, including MAPK pathways.^53^^,^^54^ Elevated plasma ceramide has also been reported in ACS,^55^ suggesting that tissue-level ceramide accumulation may be reflected systemically. While observational studies have linked blood Cer(d18:1/16:0) to cardiovascular risk,^56^^,^^57^ our Mendelian randomization analysis showed a positive association between genetically predicted plasma Cer(d18:1/16:0) and trunk fat mass, suggesting a possible relationship with central adiposity. Nevertheless, the cross-sectional design and lack of direct lipid perturbation experiments preclude determining whether local ceramide accumulation in ACS adipose tissue is a cause or consequence of inflammatory remodeling, and these processes may interact bidirectionally. The 11-DOC-associated and Cer(d18:1/16:0)-associated gene modules shared only two genes, suggesting that circulating 11-DOC and local ceramide accumulation represent complementary but partially distinct molecular features of adipose tissue dysfunction in ACS.

Periadrenal adipose tissue should be interpreted in light of its depot-specific characteristics. Although anatomically located within the visceral adipose compartment and accessible during adrenalectomy, it is not identical to typical visceral depots such as omental or mesenteric fat. Human perirenal/retroperitoneal adipose tissue can contain brown adipose tissue (BAT)-like components; UCP1-positive adipocytes have been detected in nearly half of retroperitoneal fat samples from patients undergoing surgery for benign adrenal tumors, with UCP1-positive samples showing higher expression of brown adipocyte markers, including *PGC1*, *DIO2*, and *ADRB3*.^58^ UCP1-positive adipocytes and BAT-related gene enrichment have also been reported in perirenal adipose tissue from healthy kidney donors.^59^ These thermogenic features may influence transcriptomic and lipidomic profiles related to energy and lipid metabolism. Because ACS/NFAT comparisons were performed within the same periadrenal depot, between-depot differences were minimized, but caution is required when extrapolating these findings to other visceral depots.

In conclusion, our multi-omics analysis identified circulating 11-DOC and adipose ceramide accumulation as systemic and tissue-level molecular features associated with adipose inflammation and visceral adiposity in ACS. These findings provide an integrative but hypothesis-generating view of how adrenal steroid imbalance and local lipid alterations may relate to adipose tissue remodeling in ACS, and may inform future studies aimed at improving metabolic risk stratification in this condition.

### Limitations of the study

First, this was a single-center, cross-sectional study; therefore, the clinical and tissue-level findings should be interpreted as associations rather than causal relationships. Residual confounding by comorbidities, medications, diet, physical activity, or other unmeasured factors cannot be excluded. Although NFAT was used as a clinically relevant disease control, it should not be regarded as normal adipose tissue,^60^ thus, our findings should be interpreted as ACS-associated molecular features compared with NFAT. Second, the sample sizes for tissue-based RNA-seq, lipidomics, histological, and matched multi-omics analyses were limited, and sample availability differed across data layers. These factors may reduce statistical power, increase sensitivity to outliers or subgroup effects, and introduce selection bias. In particular, the 11-DOC-VSR association was identified in a small subgroup of premenopausal women and was not significant in the overall age- and sex-adjusted model; WGCNA based on matched omics datasets was also partly sensitive to exclusion of individual samples. Third, periadrenal adipose tissue may not represent other visceral fat depots, and possible BAT-like components may influence transcriptomic and lipidomic profiles. Deconvolution analysis provides inferred cell-type proportions rather than direct cell counts. Fourth, the RAW264 experiments should be interpreted as a simplified, hypothesis-generating model. RAW264 cells are a murine macrophage cell line and do not capture the cellular diversity of adipose tissue. In addition, the 1,000 nM 11-DOC concentration exceeded physiological circulating levels,^61^ the receptor pathway mediating the transcriptional response was not experimentally defined, and comprehensive protein-level validation or functional assays were not performed. Finally, the Mendelian randomization analysis should be interpreted cautiously. In addition, BMI-adjusted waist-to-hip ratio could not be used as the outcome because outcome summary statistics were unavailable for several of the instrumental SNPs. Trunk fat mass is not a direct measure of visceral adipose tissue, leave-one-out analysis indicated sensitivity to the limited instrument set, and the European-ancestry GWAS datasets may limit direct extrapolation to our Japanese clinical cohort. Thus, the findings do not establish a visceral-fat-specific causal effect of Cer(d18:1/16:0). Functional studies—such as adipocyte-macrophage co-culture systems, pharmacological inhibition of 11-DOC signaling, and tissue-specific *in vivo* models—will be required to establish the directionality and causality of these associations.

## Resource availability

### Lead contact

Requests for further information and resources should be directed to and will be fulfilled by the lead contact, Maki Yokomoto-Umakoshi (umakoshi.maki.498@m.kyushu-u.ac.jp).

### Materials availability

This study did not generate new unique reagents.

### Data and code availability

The raw RNA-seq FASTQ files and processed gene-level count matrices generated in this study have been deposited in the Gene Expression Omnibus under accession number GSE330759. The raw lipidomics concentration data generated in this study have been deposited in Zenodo (https://doi.org/10.5281/zenodo.20337809). The plasma steroid metabolomics data analyzed in this study were originally generated in our previously published clinical study^26^ and have been deposited in Zenodo (https://doi.org/10.5281/zenodo.21555983). The analysis scripts required to reproduce the main and supplementary figures are available at: https://github.com/md108050/acs-nfat-multiomics-public. Any additional information required to reanalyze the data reported in this paper is available from the lead contact upon request.

## Acknowledgments

We thank Ms. Tomoko Nakaji for her assistance and the medical staff of the Department of Endocrinology, Metabolism, and Diabetes, Kyushu University Hospital for their support. Figure 1A and Graphical Abstract were created using BioRender (https://app.biorender.com/). We also thank Editage (https://www.editage.jp/) for English language editing.

This work was supported in part by the grants from 10.13039/501100001691Japan Society for the Promotion of Science (JSPS KAKENHI) (JP26K02833 to M.Y.-U., JP26K19538 to M.F., and JP22H04993 to Y.O.), the Clinical Research Promotion Foundation (to M.Y.-U.); the 10.13039/100007449Takeda Science Foundation (to M.Y.-U.), the 10.13039/100008732Uehara Memorial Foundation (to Y.O.), Terumo Life Science Foundation (to M.Y.-U.), and 10.13039/100009619Japan Agency for Medical Research and Development (AMED)-CREST (25gm1910011h0001 to Y.O.).

## Author contributions

Conceptualization, M.Y.-U. and Y.O.; methodology, H.N., M.Y.-U., K.N., Y.I., T.B., and H.U.; software, H.N. and M.Y.-U.; validation, H.N., M.Y.-U., K.N., and R.O.; formal analysis, H.N., M.Y.-U., and K.N.; investigation, K.N., Y.K., R.O., H.K., N.I., Y.N., T.F., T.O., H.T., T.M., Y.I., M.E., and T.B.; resources, T.M., Y.I., M.E., and T.B.; data curation, H.N., M.Y.-U., K.N., and R.O.; writing – original draft, H.N., M.Y.-U., and Y.O.; writing – review and editing, M.Y.-U., H.U., and Y.O.; visualization, H.N. and M.F.; supervision, H.U. and Y.O.; project administration, M.Y.-U.; funding acquisition, M.Y.-U., M.F., and Y.O.

## Declaration of interests

The authors declare no competing interests.

## Declaration of generative AI and AI-assisted technologies in the manuscript preparation process

During the preparation of this work, the authors did not use generative AI or AI-assisted technologies in the manuscript preparation process.

## STAR★Methods

### Key resources table

**Table:** 

REAGENT or RESOURCE	SOURCE	IDENTIFIER
**Antibodies**		

Mouse monoclonal anti-CD68 antibody, clone PG-M1	Agilent/Dako	Cat# M0876; RRID: AB_2074844
Rabbit monoclonal anti-CD204 antibody, clone EPR24403-17	Abcam	Cat# ab271070; RRID: AB_3756663

**Biological samples**		

Human periadrenal adipose tissue from patients with autonomous cortisol secretion	This paper	N/A
Human periadrenal adipose tissue from patients with non-functioning adrenal tumors	This paper	N/A
Human plasma samples from patients with autonomous cortisol secretion and non-functioning adrenal tumors	Nakao et al.^26^	N/A

**Chemicals, peptides, and recombinant proteins**		

11-deoxycorticosterone	Sigma-Aldrich	Cat# D7000

**Critical commercial assays**		

RNeasy Mini Kit	Qiagen	Cat# 74004
ISOGEN	Nippon Gene	Cat# 311-02501
PrimeScript RT reagent kit (Perfect Real Time)	Takara	Cat# RR037A
NEBNext Ultra Directional RNA Library Prep Kit for Illumina	New England BioLabs	Cat# E7760L
Cell Counting Kit-8	Dojindo	Cat# CK04

**Deposited data**		

Raw RNA-seq FASTQ files and processed gene-level count matrices generated in this study	This paper	GEO: GSE330759
Raw lipidomics concentration data generated in this study	This paper	Zenodo: https://doi.org/10.5281/zenodo.20337809
Plasma steroid metabolomics data analyzed in this study	Nakao et al.^26^	Zenodo: https://doi.org/10.5281/zenodo.21555983
Public RNA-seq dataset of omental visceral adipose tissue from patients with Cushing’s syndrome and matched controls	García-Eguren et al.^15^	GEO: GSE140126
Human white adipose tissue single-cell RNA-seq reference dataset	Emont et al.^62^	Single Cell Portal: SCP1376
GWAS summary statistics for plasma Cer(d18:1/16:0)	Surendran et al.^31^	GWAS Catalog: GCST9024534
GWAS summary statistics for trunk fat mass	Elsworth et al.^32^/UK Biobank	IEU OpenGWAS: ukb-b-20044

**Experimental models: Cell lines**		

Mouse: RAW264 cells	RIKEN BioResource Research Center	Cat# RCB0535; RRID: CVCL_4478

**Oligonucleotides**		

qPCR primers used in this study	This paper	Table S22

**Software and algorithms**		

R	R Project for Statistical Computing	https://www.r-project.org/
DESeq2 version 1.44.0	Love et al.^63^	https://bioconductor.org/packages/DESeq2/
MetaboAnalyst 6.0	Pang et al.^64^	https://www.metaboanalyst.ca/
enrichR version 3.2	Jawaid^65^	https://cran.r-project.org/package=enrichR
decoupleR version 2.10.0	Badia-i-Mompel et al.^28^	https://bioconductor.org/packages/release/bioc/html/decoupleR.html
BisqueRNA version 1.0.5	Jew et al.^66^	https://cran.r-project.org/package=BisqueRNA
WGCNA version 1.72.5	Langfelder and Horvath^29^^,^^30^	https://cran.r-project.org/package=WGCNA
SliceOmatic version 6, rev-5	Tomovision	https://www.tomovision.com/
emmeans version 1.8.9	Lenth	https://cran.r-project.org/web/packages/emmeans/index.html
Adiposoft plugin version 1.16	Galarraga et al.^67^	N/A
Fiji/ImageJ version 1.0.0	NIH	https://imagej.net/software/fiji/
QuPath version 0.5.1	Bankhead et al.^68^	https://qupath.github.io/
Multi-ChromatoAnalysT version 1.3.4.0	Beforce Co., Japan	N/A
TwoSampleMR version 0.6.17	Hemani et al.^69^	https://mrcieu.github.io/TwoSampleMR/
MR-PRESSO version 1.0	Verbanck et al.^70^	https://github.com/rondolab/MR-PRESSO
STAR version 2.7.11a	Dobin et al.	https://github.com/alexdobin/STAR
clusterProfiler version 4.12.6	Yu et al.^71^	https://bioconductor.org/packages/release/bioc/html/clusterProfiler.html
Bcl2Fastq	Illumina	N/A

**Other**		

Illumina NextSeq 500 platform	Illumina	N/A
Illumina NovaSeq X Plus platform	Illumina	N/A
Nexera X2 UHPLC system	Shimadzu	N/A
LCMS-8060 triple quadrupole mass spectrometer	Shimadzu	N/A
InertSustain C18 column	GL Sciences	N/A
EnSpire multimode plate reader	PerkinElmer	N/A
Analysis scripts for this study	This paper	https://github.com/md108050/acs-nfat-multiomics-public

### Experimental model and study participant details

#### Human participants

This retrospective cross-sectional study was conducted at Kyushu University Hospital, Japan. The study protocol was approved by the Institutional Ethics Committee (No. 21025) and performed in accordance with the clinical study guidelines of the Ministry of Health and Labor, Japan. Informed consent was obtained from all the patients upon hospital admission.

This study enrolled 206 patients admitted to our endocrine unit for the evaluation of adrenal tumors between January 2013 and September 2021 (Figure 1A). Patients with pheochromocytoma, primary aldosteronism, or adrenocortical carcinoma were not eligible for enrollment, as determined by clinical data, imaging features, and/or postoperative histopathological findings. Patients aged ≥80 years (*n* = 3) or those who had not undergone 1-mg DST (*n* = 11) were also excluded. Additionally, one patient lacking all of the following data was excluded: CT images, periadrenal adipose tissue analysis (lipidomics and RNA-seq), and plasma steroid metabolomics. Consequently, 93 patients with ACS and 98 those with NFAT were included.

Patients were not randomly assigned to groups because this was a retrospective observational study; group classification was based on predefined diagnostic criteria, and no formal sample size calculation was performed. Age and sex were obtained from medical records and are reported in Table 1; gender identity, ancestry, race, ethnicity, and socioeconomic status were not systematically recorded, which may limit generalizability beyond this single-center Japanese cohort.

#### RAW264 cells

RAW264 cells (catalog no. RCB0535; RRID: CVCL_4478), a murine macrophage cell line, were obtained from the RIKEN BioResource Research Center (Ibaraki, Japan)^72^ and cultured in Dulbecco’s Modified Eagle Medium supplemented with 10% fetal bovine serum and antibiotics at 37°C in a humidified atmosphere containing 5% carbon dioxide. The sex of the source animal was not available from the cell-line information and was not analyzed as a biological variable.

### Method details

#### Study design and diagnosis

Patients with ACS and NFAT were diagnosed based on serum cortisol levels after the DST ≥1.8 μg/dL (50 nmol/L) and <1.8 μg/dL, respectively. In this study, ACS included both CS and MACS. The diagnosis of CS was made according to the clinical criteria in the Clinical Practice Guidelines of the Endocrine Society,^73^ and MACS was defined as DST-cortisol ≥1.8 μg/dL in the absence of overt clinical signs.^74^ Clinical data, including smoking status, alcohol intake, and menopausal status, were obtained from medical records. Alcohol intake was defined as more than 3 units per day, and smoking status was defined as past or current smoking. Menopause was defined as the absence of menstruation for at least 1 year at baseline, based on an interviewer-assisted questionnaire. We further confirmed that all women classified as postmenopausal had serum follicle-stimulating hormone (FSH) levels greater than 30 mIU/mL, consistent with previously used criteria for postmenopausal status.^75^^,^^76^

#### RNA-seq of periadrenal adipose tissue

Periadrenal adipose tissue samples were collected during adrenalectomy from 14 patients with ACS and 6 with NFAT. The specimens were promptly frozen and stored at −80 °C until analysis. For RNA-seq, total RNA was extracted, and its quality and quantity were assessed. Ribosomal RNA was depleted, and RNA libraries were prepared and sequenced on the Illumina NextSeq 500 platform. The resulting paired-end sequence reads were aligned to the human reference genome (hg19), and gene expression levels were quantified.

#### Plasma steroid metabolomics

Plasma steroid metabolomics data were available for 72 patients with ACS and 85 with NFAT from our previous study.^26^ Plasma samples were collected in the early morning on the day after admission, in the fasting state, after the patients had rested for 30 min. Steroid profiling of 21 plasma metabolites was performed using liquid chromatography–tandem mass spectrometry.^26^^,^^61^^,^^77^ A mixture of 50 μL of plasma, 500 μL of methanol, and 10 μL of an internal standard solution containing stable isotope–labeled compounds in methanol was vortexed for 1 min, sonicated for 5 min, and centrifuged at 16,000 × g for 5 min at 4 °C to remove proteins. The supernatant (500 μL) was diluted with 1,500 μL of water/formic acid (100/0.1, v/v) prior to solid-phase extraction. The Oasis HLB cartridges (1 cc, 30 mg; Waters, Milford, MA, USA) were preconditioned with methanol and water (each containing 0.1% formic acid), washed sequentially with water/formic acid (100/0.1, v/v), ethanol/water/formic acid (15/85/0.1, v/v/v), and hexane, and analytes were eluted with 1 mL of methanol; the eluate was evaporated to dryness in a centrifugal evaporator and reconstituted in 60 μL of water/methanol (1/1, v/v). The LC/MS system consisted of a Nexera X2 UHPLC system coupled to an LCMS-8060 triple-quadrupole mass spectrometer (Shimadzu, Kyoto, Japan), with chromatographic separation on an InertSustain C18 column (2.1 mm i.d. × 150 mm, 3 μm; GL Sciences, Tokyo, Japan). The column was maintained at 50 °C, with a flow rate of 0.3 mL/min and an injection volume of 10 μL. Mobile phase A was 5 mM ammonium acetate in water/acetonitrile (75/25, v/v) and mobile phase B was 5 mM ammonium acetate in 2-propanol; the gradient program was as follows: 1% B, 0 min; 1–38% B, 0–17 min; 38–99% B, 17–25 min; 99% B, 25–35 min; 99–1% B, 35–36 min; and 1% B, 36–46 min. Multiple reaction monitoring was used for steroid quantification. Identification and quantification of the steroid molecules were performed using Multi-ChromatoAnalysT (ver.1.3.4.0, Beforce Co., Japan).

#### Lipidomics of periadrenal adipose tissue

Lipidomics was performed on periadrenal adipose tissue collected and stored similar to RNA-seq, from 13 patients with ACS and 9 with NFAT. Pretreatment of adipose tissue samples was performed according to a published protocol;^78^ tissue was homogenized using a Multi-Beads Shocker (Yasui Kikai, Osaka, Japan) at 3,000 rpm for 5 s under liquid nitrogen, mixed with methanol containing stable isotope–labeled internal standards, vortexed for 1 min, sonicated for 5 min, and centrifuged at 16,000 × g for 5 min at 4 °C, and the methanol supernatant (400 μL) was collected. Lipid extraction and sample preparation were carried out using a published method:^79^ lipids were extracted by a modified Bligh and Dyer procedure with methanol/chloroform/water (5:2:2, v/v/v) supplemented with a stable isotope–labeled lipid internal standard mixture (SPLASH Lipidomix Mass Spec Standard, Avanti Polar Lipids, Alabaster, AL, USA), and lipidomic analyses were performed using supercritical fluid chromatography–mass spectrometry (SFC/MS).^80^ The SFC/MS system consisted of a Nexera UC supercritical fluid chromatography system coupled to an LCMS-8060NX triple-quadrupole mass spectrometer (Shimadzu, Kyoto, Japan). Separation was performed on an ACQUITY UPC^2^ Torus diethylamine column (3.0 mm i.d. × 100 mm, 1.7 μm; Waters, Milford, MA, USA) at 50 °C, using supercritical CO_2_ as mobile phase A and methanol/water (95/5, v/v) containing 0.1% (w/v) ammonium acetate as mobile phase B (modifier). The mobile-phase flow rate was 1.0 mL/min, the make-up pump (same composition as the modifier) flow rate was 0.1 mL/min, the back pressure regulator was set to 10 MPa, and the injection volume was 2 μL. The modifier gradient was as follows: 1% B, 0–1 min; 1–75% B, 1–24 min; 75% B, 24–26 min; and 1% B, 26–30 min. Multiple reaction monitoring was used for lipid quantification. Identification and quantification of the lipid molecular species were performed using Multi-ChromatoAnalysT (ver.1.3.4.0, Beforce Co., Japan). This platform was used to quantify lipid species across five lipid classes: fatty acyls, glycerolipids, glycerophospholipids, sphingolipids, and sterol lipids.

#### Multi-omics data analysis

##### Data preprocessing

Each analysis was performed in all patients for whom the required clinical data or biospecimens of adequate quality were available. Integrative analyses requiring paired data were restricted to matched samples from the same individuals. For RNA-seq of periadrenal adipose tissue, gene expression data were normalized using the DESeq2 package (version 1.44.0),^63^ with variance-stabilizing transformation applied prior to downstream analysis. For plasma steroid metabolomics and lipidomics of periadrenal adipose tissue, data preprocessing was performed using MetaboAnalyst 6.0.^64^ For each metabolite or lipid feature, missing values were imputed with one-fifth of the minimum positive value observed for that feature. The imputed data were then normalized by the median value of each sample and log-transformed. Plasma steroid metabolomics data were additionally scaled by mean-centering and division by the standard deviation of each variable, whereas lipidomics data were not additionally autoscaled after log transformation. For subgroup-specific steroid association analyses, plasma steroid metabolomics data were preprocessed separately within premenopausal women, postmenopausal women, and men. For WGCNA-based integration with RNA-seq, matched samples were extracted from the steroid metabolomics dataset preprocessed without subgroup stratification. Downstream analyses were performed using these preprocessed data.

##### RNA-seq of periadrenal adipose tissue

Differential gene expression analysis was conducted using DESeq2 (version 1.44.0).^63^ Genes with |logFC| > 1 and FDR <0.05 were defined as differentially expressed genes (DEGs). To visualize transcriptomic differences between ACS and NFAT groups, a UMAP plot was generated using the normalized expression data. Pathway enrichment analyses of identified DEGs were performed using the enrichR package (version 3.2).^65^ Gene set libraries included the Molecular Signatures Database Hallmark, Gene Ontology Biological Process (GOBP), and the NCI-Nature Pathway Interaction Database (2016 release). Transcription factor and pathway activities were inferred using the decoupleR package (version 2.10.0).^28^

To compare the adipose tissue transcriptomic signature in our cohort with an independent overt CS dataset, we used the public RNA-seq dataset (GEO: GSE140126),^15^ which includes omental visceral adipose tissue samples from patients with CS (*n* = 6) and matched controls (*n* = 12). Each dataset was analyzed independently using DESeq2, and pathway-level concordance was assessed by gene set enrichment analysis using MSigDB Hallmark gene sets. Normalized enrichment scores were compared between our ACS vs. NFAT signature and the public overt CS vs. control signature. Because our ACS group included both CS and MACS, a MACS-only vs. NFAT comparison was also performed.

##### Deconvolution analysis

To investigate immune cell composition differences between ACS and NFAT groups, deconvolution analysis was performed using the BisqueRNA package (version 1.0.5).^66^ As a reference dataset, we used publicly available single-cell RNA-seq data of human white adipose tissue (Single Cell Portal: SCP1376),^62^ including 166,149 cells from 32 samples. Because SCP1376 includes multiple adipose depots, we restricted the reference to omental visceral adipose tissue, resulting in 59,802 reference cells. Cell-type proportions were estimated for 17 annotated cell types: adipocytes, adipose stem/progenitor cells, B cells, dendritic cells, endothelial cells, hMac1, hMac2, hMac3, lymphatic endothelial cells, mast cells, mesothelium, monocytes, neutrophils, natural killer cells, pericytes, smooth muscle cells, and T cells. For each annotated cell type, up to 500 cells were randomly downsampled to reduce imbalance in reference cell numbers across cell types and to avoid overrepresentation of highly abundant cell populations. A reference expression matrix was generated and used for deconvolution of bulk RNA-seq data. Estimated RNA-based proportions of these cell groups were compared between ACS and NFAT using the Mann–Whitney U test.

##### Plasma steroid metabolomics

We evaluated correlations between individual steroid metabolite levels and VSR using Spearman’s rank correlation. To account for sex- and age-related differences in steroid profiles,^81^ patients were subclassified as premenopausal women (ACS, *n* = 17; NFAT, *n* = 12), postmenopausal women (ACS, *n* = 26; NFAT, *n* = 34), and men (ACS, *n* = 29; NFAT, *n* = 39). In this analysis, FDR <0.1 was considered statistically significant. Multiple linear regression analysis was subsequently performed with VSR as the dependent variable and each steroid metabolite as the independent variable. Covariates included serum cortisol levels, body mass index (BMI), smoking status, and alcohol intake. As a sensitivity analysis, the metabolite–VSR association identified in the subgroup analysis was also evaluated in the overall ACS cohort using the same multivariable regression model.

##### Lipidomics of periadrenal adipose tissue

Fold changes in lipid concentrations between ACS and NFAT groups were calculated on a logarithmic scale, expressed as logFC. FDR <0.1 was considered statistically significant. PLS-DA was used to determine whether ACS exhibited distinct lipidomic profiles in periadrenal adipose tissue. VIP scores >1 were used to identify lipid features with above-average contribution to group discrimination in the PLS-DA model.

##### WGCNA

WGCNA was performed using variance-stabilized RNA-seq expression values. Separate WGCNA analyses were conducted for samples with matched RNA-seq and plasma steroid metabolomics data and for samples with matched RNA-seq and lipidomics data. In each analysis, genes were ranked by their expression variance across the matched samples included in that analysis, and the top 500 most variable genes were selected for network construction. The soft-thresholding power was selected using the scale-free topology criterion implemented in the WGCNA package (version 1.72.5).^29^^,^^30^ An unsigned network was constructed, modules were detected by hierarchical clustering and dynamic tree cutting, and module eigengenes were correlated with steroid metabolites or lipid features using Pearson’s correlation. Leave-one-out sensitivity analyses were performed to assess the sensitivity of WGCNA module–metabolite correlations to individual samples. One sample was excluded at a time, the WGCNA network was reconstructed using the remaining matched samples, and the correlation between the module eigengene of interest and the corresponding metabolite was recalculated. Pathway enrichment analysis for relevant modules was performed with the enrichR package (version 3.2),^65^ using the Hallmark and GOBP gene set libraries.

#### Body composition evaluation

CT images at the level of the third lumbar vertebra (L3), obtained between the initial visit and diagnostic confirmation, were used to assess body composition. SliceOmatic software (version 6, rev-5; Tomovision, Canada) quantified visceral fat area (VFA) and subcutaneous fat area (SFA). Analyses were performed in a blinded manner using default software settings. Intra- and interobserver reliability were assessed in 30 randomly selected subjects. Intraobserver reliability: VFA (Intraclass Correlation Coefficient [ICC] = 1.00), SFA (ICC = 0.99). Interobserver reliability: VFA (ICC = 0.99), SFA (ICC = 0.94), all showing excellent reliability (ICC ≥0.90). The VSR was calculated by dividing VFA by SFA. Group comparisons of VSR between ACS and NFAT were evaluated using analysis of covariance, adjusting for age, sex, BMI, alcohol intake, and smoking status. Adjusted means with 95% CI were estimated using the emmeans package in R (version 1.8.9). To account for skewness, VSR was log-transformed prior to modeling, and adjusted means were back-transformed to the original scale for presentation.

#### Adipocyte size quantification

Adipocyte size was quantified from HE-stained serial 4-μm-thick sections of periadrenal adipose tissue obtained from four patients with ACS and three with NFAT, using fresh frozen sections. Image analysis was performed with the Adiposoft plugin (version 1.16)^67^ in Fiji (ImageJ version 1.0.0) in automated mode. Minimum and maximum diameters were set to 20 μm and 200 μm, respectively. For each sample, a representative field was selected for analysis. Data were summarized as the median adipocyte area and diameter per sample.

#### Immunohistochemistry

Immunohistochemistry was performed using FFPE and OCT-embedded frozen sections of periadrenal adipose tissue. FFPE immunohistochemistry for CD68 and CD204 was performed in six ACS cases and one available NFAT case. OCT-embedded frozen sections were used for supplementary CD204 quantification in ACS (*n* = 4) and NFAT (*n* = 3) cases. The FFPE and OCT/frozen samples were obtained from different cases. Serial 4-μm-thick sections were cut and stained with anti-CD68 (1:100 dilution, Agilent, catalog no. M0876, RRID: AB_2074844) and anti-CD204 antibodies. The anti-CD204 antibody used was a rabbit monoclonal antibody (clone EPR24403-17, dilution 1:1000, Abcam, catalog no. ab271070, RRID: AB_3756663). Representative high-magnification images were exported using QuPath (version 0.5.1).^68^ For supplementary quantitative analysis of OCT/frozen CD204 staining, the entire available snapshot area was annotated as one region of interest (ROI) per sample, and CD204-positive cells were detected using QuPath based on 3,3′-diaminobenzidine staining intensity. CD204-positive cell density was calculated as the number of positive cells per mm^2^.

#### Biochemical measurements

Morning serum cortisol, ACTH, and DHEA-S were measured using samples collected in the early morning on the day after admission, in the fasting state, after the patients had rested for 30 min. For DST-cortisol, 1 mg of dexamethasone was administered at 23:00 h the day before sample collection, and serum cortisol was measured the following morning under the same fasting and resting conditions. Serum cortisol, ACTH, DHEA-S, and other routine biochemical measurements were obtained from clinical laboratory records at Kyushu University Hospital. Serum FSH levels were measured using a chemiluminescent enzyme immunoassay (TOSOH, Japan).

#### Mendelian randomization

To investigate the potential causal effect of ceramide on adiposity, we performed a two-sample Mendelian randomization analysis. Six SNPs associated with plasma Cer(d18:1/16:0) levels at genome-wide significance (*p* < 5 × 10^−8^) were selected as instrumental variables using linkage disequilibrium clumping (r^2^ < 0.001, 10,000-kb window) from a GWAS of 19,994 individuals of European ancestry.^31^ Data were downloaded from the GWAS Catalogue.^82^ F-statistics suggested that these variants strongly predicted plasma Cer(d18:1/16:0) levels (F > 10). Outcome data on trunk fat mass (measured by bioelectrical impedance) were derived from 454,588 European individuals in the UK Biobank.^32^ BMI-adjusted waist-to-hip ratio was initially considered as the outcome; however, outcome summary statistics were unavailable for several of the instrumental SNPs, and trunk fat mass was therefore used as the outcome instead. The primary analysis used the IVW method under a multiplicative random-effects model. Heterogeneity was assessed using Cochran’s Q test. As sensitivity analyses, we used the weighted median method (robust up to 50% invalid instruments) and MR-Egger regression (to assess potential directional pleiotropy via the intercept). We also performed leave-one-out analysis to assess whether the association was disproportionately influenced by any single SNP. To evaluate non-lipid traits potentially relevant to horizontal pleiotropy, PheWAS associations were examined for all instrumental SNPs. To detect and correct for horizontal pleiotropy, we applied MR-PRESSO analysis, which identifies and removes outlier SNPs. The analysis relied on the three core instrumental variable assumptions: (i) the genetic variants are associated with the exposure (relevance), supported by genome-wide significance and F-statistics >10; (ii) the variants are independent of confounders of the exposure–outcome association (independence); and (iii) the variants affect the outcome only through the exposure (exclusion restriction), which was examined using MR-Egger regression, PheWAS of the instrumental variants, and MR-PRESSO. Both the exposure and outcome summary statistics were derived from individuals of European ancestry, and participant overlap between the two samples was not expected, although it could not be formally quantified from summary-level data. Because only summary-level data were used, covariate adjustment could not be harmonized across the two source samples. No *a priori* power calculation was performed, and because a single exposure–outcome pair was tested, no adjustment for multiple comparisons was applied. Bidirectional Mendelian randomization was not performed, and this analysis was not pre-registered. Because the analysis used only publicly available summary-level GWAS data, no additional ethics approval or participant consent was required. Analyses were performed using the TwoSampleMR (version 0.6.17)^69^ and MR-PRESSO (version 1.0)^70^ packages.

#### *In vitro* experiment

For experiments, cells were seeded at 2.0 × 10^5^ cells/well in 12-well plates (1 mL medium per well) one day before treatment. Cells were then treated with either vehicle (0.1% dimethyl sulfoxide) as control or 11-deoxycorticosterone (11-DOC; Sigma-Aldrich, catalog #D7000) at a final concentration of 1000 nM for 48 h. Each group consisted of three independent wells (*n* = 3). Total RNA was extracted using the RNeasy Mini Kit (Qiagen, catalog #74004). Culture medium was aspirated, cells were washed with phosphate-buffered saline, and then lysed with ISOGEN (Nippon Gene, catalog #311-02501). RNA was purified according to the manufacturer’s protocol and eluted in 20 μL of RNase-free water. RNA concentration and purity were assessed using a NanoDrop spectrophotometer, with A260/A280 ratios of 2.0–2.05. Total RNA (500 ng per sample) was reverse transcribed using the PrimeScript RT reagent kit (Perfect Real Time) (Takara, catalog #RR037A). Libraries for RNA-seq were prepared using the NEBNext Ultra Directional RNA Library Prep Kit for Illumina (New England BioLabs, catalog #E7760L) and sequenced on the NovaSeq X Plus (Illumina). Base call files were converted to the FASTQ format using Bcl2Fastq (Illumina). All reads were aligned to the mouse reference genome (mm10) using STAR (version 2.7.11a), and gene expression was quantified. Differential gene expression and pathway enrichment analyses were performed using the clusterProfiler package (version 4.12.6),^71^ with the Hallmark and GOBP gene set libraries.

Cell viability after 11-DOC treatment was assessed using CCK-8 (Dojindo, #CK04, Kumamoto, Japan). RAW264 cells were seeded in 96-well plates at 1 × 10ˆ4 or 2 × 10ˆ4 cells/well and treated with vehicle or 1,000 nM 11-DOC for 48 h. After addition of CCK-8 reagent, absorbance at 450 nm was measured at 10, 30, and 60 min using an EnSpire multimode plate reader (PerkinElmer, Waltham, MA, USA). Comparisons were performed using two-sided Welch’s *t* test within each cell density and detection time. qPCR validation was performed using RNA from RAW264 cells treated with vehicle or 11-DOC at 100 or 1000 nM for 48 h. Relative gene expression was calculated from ΔCt values and normalized to the mean value of the control group for each gene. *p* values were calculated using two-sided Welch’s *t* test on ΔCt values. Primer sequences are listed in Table S22.

### Quantification and statistical analysis

#### Statistics

Statistical analyses were performed using R (version 4.4.0). The specific test used for each analysis is described in the corresponding subsection of METHOD DETAILS and stated in each figure legend. Baseline characteristics were compared using the Mann–Whitney U test for continuous variables and Fisher’s exact test for categorical variables; adipocyte diameter and deconvolution-derived cell-type proportions were compared using the Mann–Whitney U test. All tests were two-tailed. Unless otherwise specified, asterisks in figures indicate *p* < 0.05; in Figures 3B, 4A, and 4C, asterisks indicate FDR <0.1 (Benjamini–Hochberg). For transcriptome-wide differential gene expression analyses based on RNA-seq, FDR <0.05 was used. For plasma steroid metabolomics and adipose tissue lipidomics, FDR <0.1 was used because these analyses were based on predefined metabolite or lipid panels with fewer measured features than transcriptome-wide analyses, while the availability of matched human clinical samples was limited.

Exact sample sizes and what n represents are reported in the Results, Table, and Figure legends. For clinical, histological, and immunohistochemical analyses, n represents the number of patients or biological samples, as indicated. For *in vitro* experiments, n represents the number of independent wells or biological replicates unless otherwise specified. Non-parametric tests were used where appropriate because many clinical and omics variables were not assumed to follow a normal distribution and some tissue-based analyses had limited sample sizes. This study is reported in accordance with the STROBE guideline for observational studies, and the Mendelian randomization component in accordance with the STROBE-MR guideline.

## Supplemental information

Document S1. Figures S1–S4

Table S1. Complete list of study patients, related to Figure 1

Table S2. Entire list of differentially expressed genes in periadrenal adipose tissue, related to Figure 2

Table S3. Hallmark gene set comparison with an independent CS adipose tissue dataset, related to Figure 2

Table S4. Estimated cell type proportion using deconvolution analysis, related to Figure 2

Table S5. Correlation between plasma steroid metabolites and VSR in patients with ACS, related to Figure 3

Table S6. Subgroup-specific and overall sensitivity multivariable regression analyses of steroid metabolites associated with VSR in patients with ACS, related to Figure 3

Table S7. Correlation between plasma steroid metabolites and VSR in patients with NFAT, related to Figure 3

Table S8. Entire list of genes in each module identified by WGCNA of periadrenal adipose tissue RNA-seq with matched plasma steroid metabolite data, related to Figure 3

Table S9. Leave-one-out sensitivity analysis of WGCNA module stability for steroid metabolite traits, related to Figure 3

Table S10. Enrichment analysis results of the turquoise module identified by WGCNA, related to Figure 3

Table S11. Entire list of differentially expressed genes in RAW264 cells treated with 11-DOC versus vehicle control (*in vitro*), related to Figure 3

Table S12. Enrichment analysis of upregulated DEGs in RAW264 cells treated with 11-DOC versus vehicle control (*in vitro*), related to Figure 3

Table S13. Comparison of lipid classes in periadrenal adipose tissue between ACS and NFAT, related to Figure 4

Table S14. VIP scores for each lipid class in periadrenal adipose tissue from PLS-DA, related to Figure 4

Table S15. Comparison of sphingolipid species in periadrenal adipose tissue between ACS and NFAT, related to Figure 4

Table S16. VIP scores for each sphingolipid species in periadrenal adipose tissue from PLS-DA, related to Figure 4

Table S17. Comparison of lipid species other than sphingolipids in periadrenal adipose tissue between ACS and NFAT, related to Figure 4

Table S18. Entire list of genes in each module identified by WGCNA of periadrenal adipose tissue RNA-seq with matched lipidomics data, related to Figure 5

Table S19. Leave-one-out sensitivity analysis of WGCNA module stability for sphingolipid traits, related to Figure 5

Table S20. Enrichment analysis results of the indigo module identified by WGCNA, related to Figure 5

Table S21. Mendelian randomization analysis of genetically predicted Cer(d18:1/16:0) with trunk fat mass, related to Figure 5

Table S22. qPCR primers, related to Figure 3
