# Supplementary material for: Multi-omics profiling reveals molecular features of visceral adiposity in autonomous cortisol secretion
Source: iScience. 2026 Oct 8;29(11):117776. doi: 10.1016/j.isci.2026.117776 (PMC13677310; doi:10.1016/j.isci.2026.117776)
Supplement: Supplementary file 1 [file mmc1.pdf]

iScience, Volume *29*

Supplemental information

Multi-omics profiling reveals molecular features of visceral adiposity in autonomous cortisol secretion

Hiroshi Nakao, Maki Yokomoto-Umakoshi, Masamichi Fujita, Kohta Nakatani, Yuya Kitamura, Ryo Ogasawara, Hiroki Kaneko, Norifusa Iwahashi, Yui Nakano, Tazuru Fukumoto, Tatsuki Ogasawara, Hiroki Takayanagi, Takashi Matsumoto, Yoshihiro Izumi, Masatoshi Eto, Takeshi Bamba, Hironobu Umakoshi, and Yoshihiro Ogawa

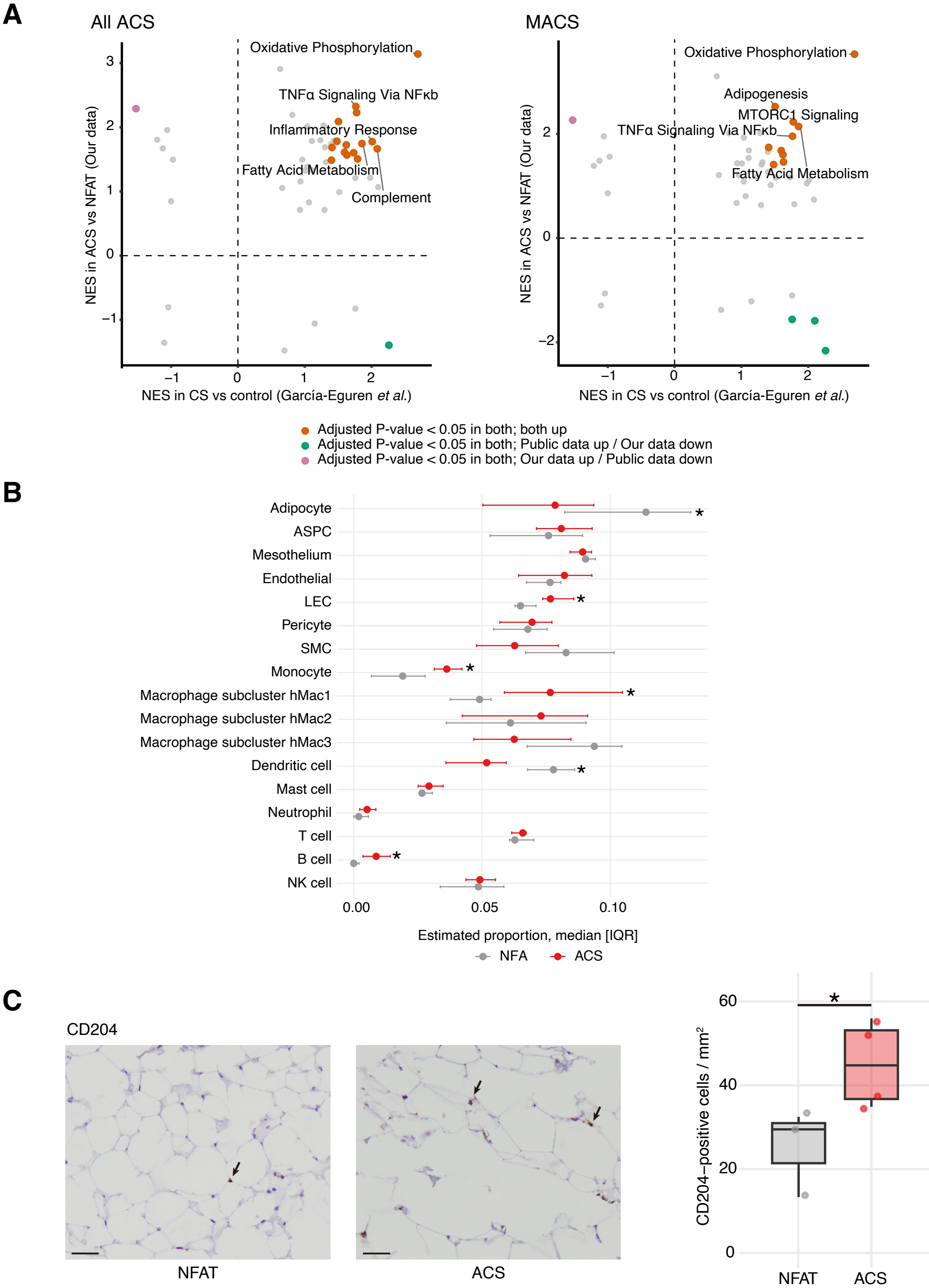

Figure S1. Comparison with a public RNA-seq dataset and supplementary analyses of macrophage-related signals in periadrenal adipose tissue. Related to Fig. 2.

(A) NES values of MSigDB Hallmark gene sets were compared between our cohort and a public RNA-seq dataset from García-Eguren et al. (CS, n = 6; matched controls, n = 12). Left, all ACS (n = 14) vs NFAT (n = 6); right, MACS (n = 9) vs NFAT (n = 6). Each point represents one gene set. Colored points indicate gene sets with adjusted P-value < 0.05 in both datasets; gray points indicate the others. Selected concordantly upregulated gene sets are labeled. (B) Estimated proportions of 17 annotated cell types inferred by deconvolution using scRNA-seq reference data (ACS, n = 14; NFAT, n = 6). Points indicate medians, and horizontal lines indicate interquartile ranges. P values were calculated using the Mann–Whitney U test (*, P < 0.05). (C) Representative images and quantification of CD204 immunohistochemistry in OCT-embedded frozen periadrenal adipose tissue sections from ACS (n = 4) and NFAT (n = 3) cases. The entire available snapshot area was annotated as one ROI per sample, and CD204-positive cell density was calculated as positive cells per mm². Each dot represents one patient. Scale bars, 50 μm. P value was calculated using the Kruskal–Wallis test (*, P < 0.05).

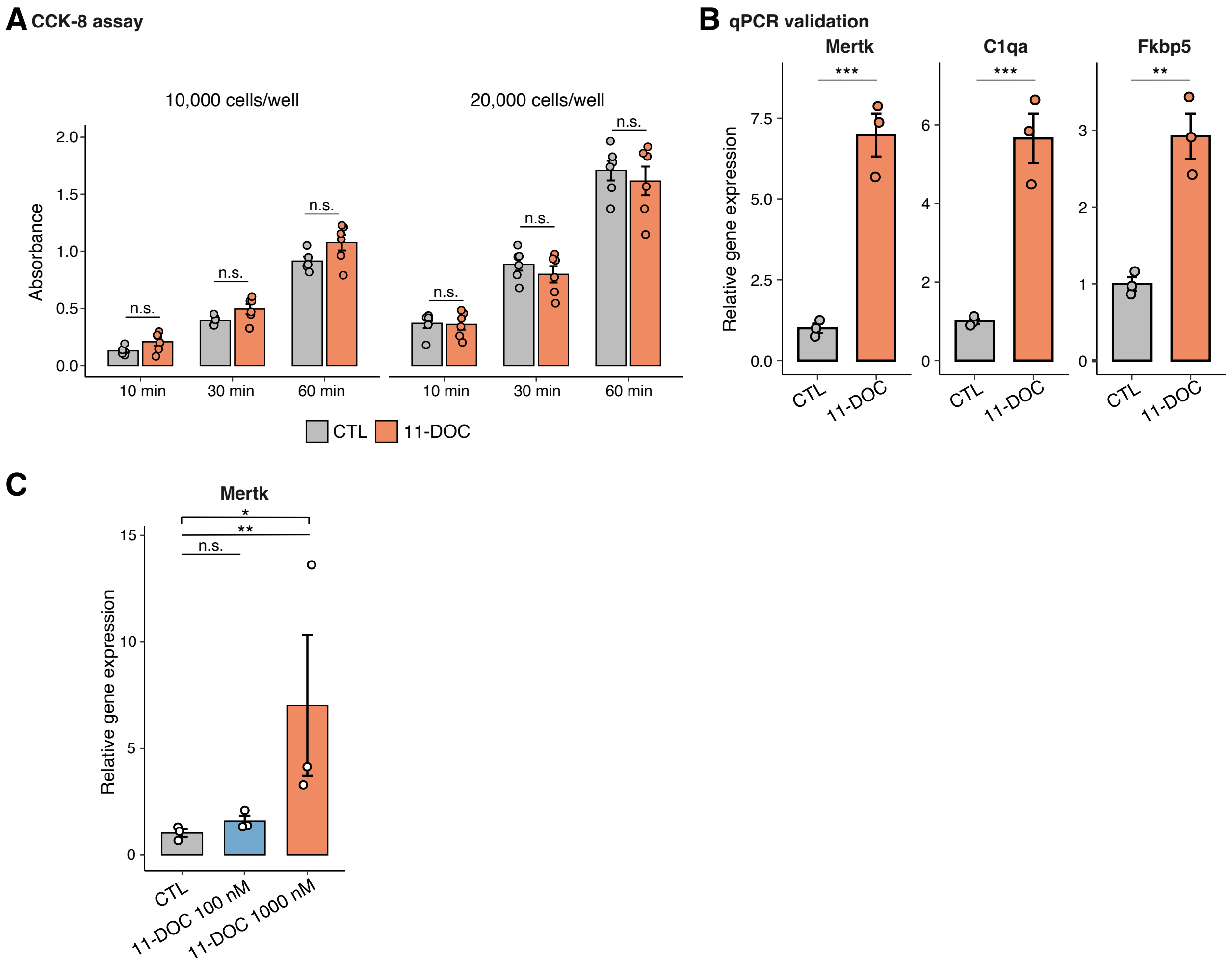

Figure S2. Validation of RAW264 cell responses to 11-DOC. Related to Fig. 3.

(A) CCK-8 assay of RAW264 cells treated with vehicle or 1,000 nM 11-DOC for 48 h. Absorbance was measured 10, 30, and 60 min after CCK-8 reagent addition (control, n = 5; 11-DOC, n = 6). (B) qPCR validation of representative 11-DOC-responsive genes after 1,000 nM 11-DOC treatment for 48 h (n = 3 per group). Relative expression was calculated from ΔCt values and normalized to the CTL mean for each gene. Data are mean ± SEM with individual values overlaid. P values were calculated using two-sided Welch's t-test. n.s., not significant; $^{**}P < 0.01$; $^{***}P < 0.001$. (C) RAW264 cells were treated with 11-DOC at 100 or 1,000 nM for 48 h (n = 3 per group). Mertk expression was normalized to S18 and is shown relative to the CTL group. Statistical comparisons were performed using ΔCt values. One-way ANOVA was used to test overall group differences, followed by Dunnett's test versus CTL. $^{*}P < 0.05$; n.s., not significant.

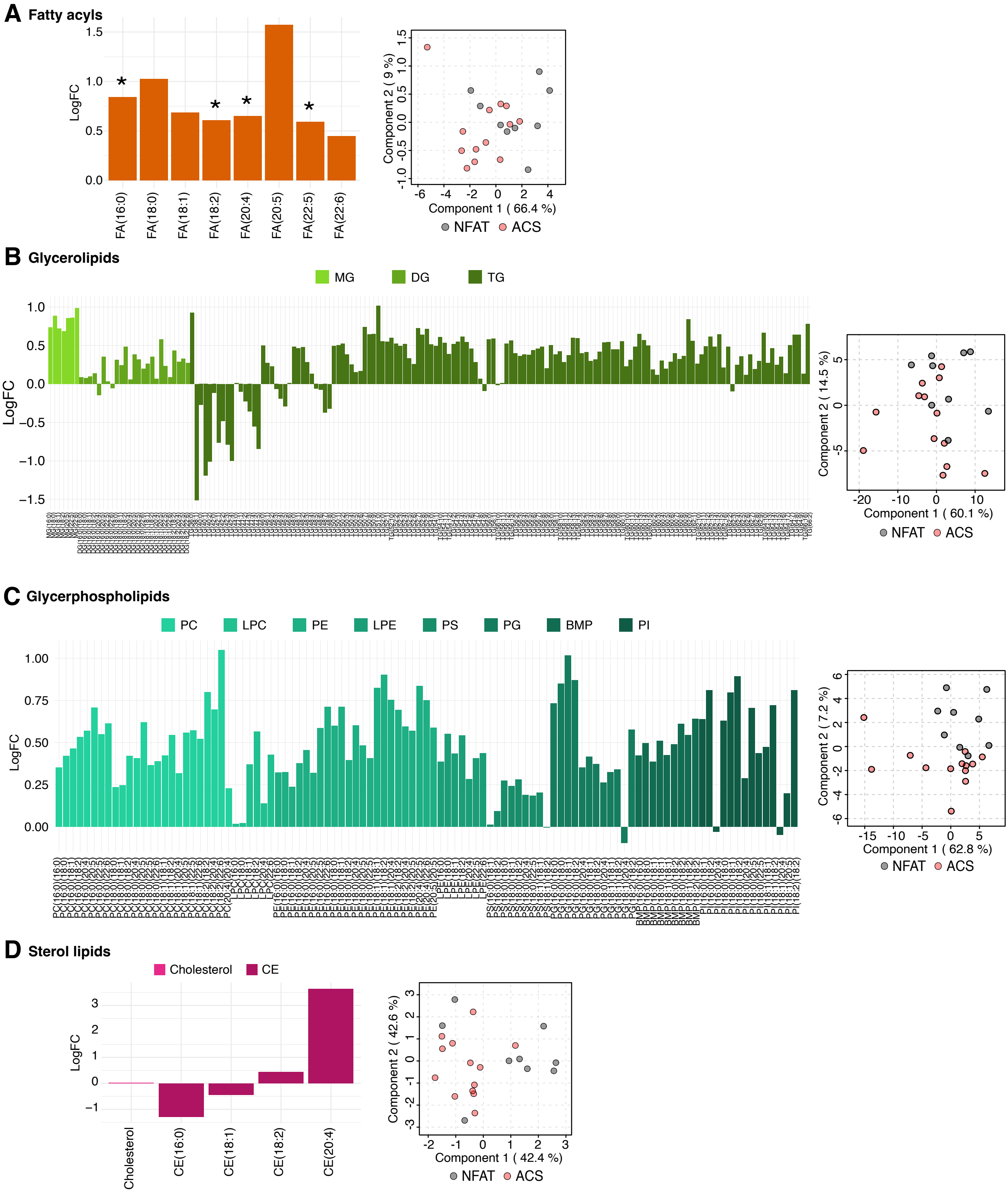

Figure S3. Lipid species profiles in non-sphingolipid classes. Related to Fig. 4.

(A) Log fold changes and PLS-DA score plot of fatty acyl species in ACS and NFAT. Asterisks indicate FDR < 0.1. (B) Log fold changes and PLS-DA score plot of glycerolipid species, including monoacylglycerols (MG), diacylglycerols (DG), and triacylglycerols (TG). (C) Log fold changes and PLS-DA score plot of glycerophospholipid species, including PC, LPC, PE, LPE, PS, PG, BMP, and PI. (D) Log fold changes and PLS-DA score plot of sterol lipid species, including cholesterol and cholesteryl esters (CE). All panels show ACS (n = 13) and NFAT (n = 9).

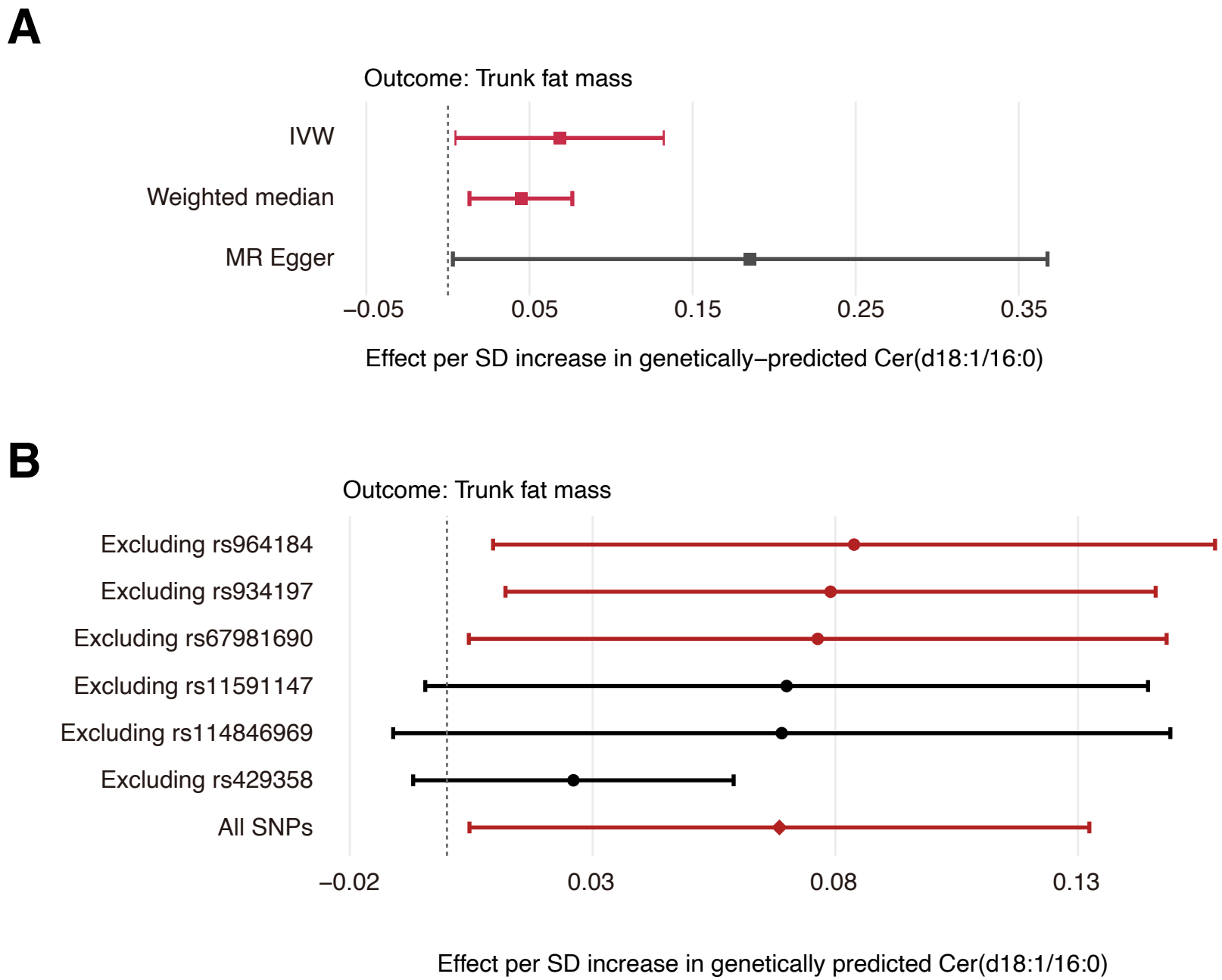

Figure S4. Mendelian randomization analyses of Cer(d18:1/16:0). Related to Fig. 5.
(A) Forest plot of Mendelian randomization analysis for plasma Cer(d18:1/16:0) levels and trunk fat mass (using all SNPs before outlier correction). Points indicate effect estimates and error bars represent 95% confidence intervals. (B) Leave-one-out analysis. Points indicate effect estimates, and horizontal lines indicate 95% confidence intervals after excluding each SNP.
